# A quantitative model for the rate-limiting process of UGA alternative assignments to stop and selenocysteine codons

**DOI:** 10.1371/journal.pcbi.1005367

**Published:** 2017-02-08

**Authors:** Yen-Fu Chen, Hsiu-Chuan Lin, Kai-Neng Chuang, Chih-Hsu Lin, Hsueh-Chi S. Yen, Chen-Hsiang Yeang

**Affiliations:** 1 Institute of Molecular Biology, Academia Sinica, Taipei, Taiwan; 2 Genome and Systems Biology Degree Program, National Taiwan University and Academia Sinica, Taipei, Taiwan; 3 Institute of Statistical Science, Academia Sinica, Taipei, Taiwan; University of Pennsylvania, UNITED STATES

## Abstract

Ambiguity in genetic codes exists in cases where certain stop codons are alternatively used to encode non-canonical amino acids. In selenoprotein transcripts, the UGA codon may either represent a translation termination signal or a selenocysteine (Sec) codon. Translating UGA to Sec requires selenium and specialized Sec incorporation machinery such as the interaction between the SECIS element and SBP2 protein, but how these factors quantitatively affect alternative assignments of UGA has not been fully investigated. We developed a model simulating the UGA decoding process. Our model is based on the following assumptions: (1) charged Sec-specific tRNAs (Sec-tRNA^Sec^) and release factors compete for a UGA site, (2) Sec-tRNA^Sec^ abundance is limited by the concentrations of selenium and Sec-specific tRNA (tRNA^Sec^) precursors, and (3) all synthesis reactions follow first-order kinetics. We demonstrated that this model captured two prominent characteristics observed from experimental data. First, UGA to Sec decoding increases with elevated selenium availability, but saturates under high selenium supply. Second, the efficiency of Sec incorporation is reduced with increasing selenoprotein synthesis. We measured the expressions of four selenoprotein constructs and estimated their model parameters. Their inferred Sec incorporation efficiencies did not correlate well with their SECIS-SBP2 binding affinities, suggesting the existence of additional factors determining the hierarchy of selenoprotein synthesis under selenium deficiency. This model provides a framework to systematically study the interplay of factors affecting the dual definitions of a genetic codon.

## Author summary

The “code book” of protein translation maps 4^3^ = 64 triplets of RNA sequences (codons) into 20 canonical amino acids and the stop signal. This code book is universal in almost all organisms on earth. Selenoproteins consist of selenium-containing amino acids–selenocysteines (Sec)–that are not among the 20 canonical amino acids. The cells “borrow” a stop codon UGA to translate selenocysteines. Since UGA maps to two possible outcomes, the translation machinery can synthesize both full-length selenoproteins (when UGA encodes selenocysteine) and truncated peptide chains (when UGA encodes translational termination). Despite extensive study about selenoprotein synthesis mechanisms, a quantitative model for how cells allocate resources to synthesize each species is yet to appear. We propose a quantitative model that can explain the dependency of experimental observables such as protein stability and Sec incorporation efficiency by various factors such as selenium concentration and mRNA levels. Saturation of those quantities implies the existence of limiting factors such as mRNA transcripts and Sec-specific tRNAs. The match between model simulations and experimental data suggests that the cellular decision making of synthesizing the two species of proteins may follow simple first-order kinetics.

## Introduction

Stop codons can be reassigned to encode amino acids [1, 2]. Failures in stop codon reassignment leads to the production of prematurely terminated proteins [3, 4], but how cellular factors influence alternative definitions of stop codons is not fully understood. While some stop codon reassignments are confined to certain species or organelles, redefinition of UGA to selenocysteine (Sec) in selenoprotein synthesis occurs in all three domains of life [5]. Selenoproteins are proteins that contain the Sec amino acid residue. Translating UGA to Sec requires Sec-tRNA^Sec^ (Sec-specific tRNA charged with Sec), the Sec insertion sequence (SECIS) element at the 3’ untranslated region (3’UTR) of selenoprotein mRNAs [4, 6, 7], and other regulatory factors such as SBP2 [8–10] and EFSec [11, 12]. Failed UGA to Sec decoding results in translation termination, with UGA being recognized by a release factor (RF) instead. RFs trigger the hydrolysis of ester bonds in peptidyl-tRNA and corresponding release of translated proteins from the ribosome [13, 14].

Translating UGA to Sec is inefficient [15–17] and influenced by the abundance of selenoprotein mRNA, Sec-tRNA^Sec^, selenium, SBP2 and the intrinsic properties of SECIS elements [8, 17–21]. Overexpression of selenoprotein mRNA reduces UGA-to-Sec decoding [18, 22], but this effect could be rescued by co-expression of uncharged Sec-specific tRNA (tRNA^Sec^) [18, 22] or SBP2 [8]. The efficiency of Sec incorporation has been shown to be positively correlated with tRNA^Sec^ or selenium supply in cells [20] yet differs among seleoproteins [23]. There are at least 25 selenoproteins in the human proteome [24] and their difference in Sec incorporation efficiency leads to a “selenoprotein hierarchy” under selenium deficiency [23]: proteins with higher Sec incorporation efficiency exploit more Sec-tRNA^Sec^ and are more rapidly synthesized. It is well known that hierarchical selenoprotein expression depends on the SECIS-SBP2 interaction [8], but whether this interaction is the sole determinant for selenoprotein hierarchy remains unclear.

Despite the aforementioned rich studies in selenoprotein translation, a systematic and quantitative characterization of the joint effects of various regulatory factors has not yet been reported. To fill this gap, we developed a simple mechanistic model that captures the quantitative characteristics of the UGA translation process and applied this model to experimental data to investigate how various regulatory factors influence the definition of UGA. We utilized differential protein half-lives from full-length and truncated selenoproteins, retrieved from a single-cell-based global protein stability (GPS) assay [25], to infer UGA definitions under cell culture conditions with variations in selenium supply and selenoprotein expression levels, and used those inferred quantities to estimate the model parameters. We found that the qualitative behavior of selenoprotein translation derived from our model closely resembles that from experimental data. Moreover, we re-capitulated the selenoprotein hierarchy by measuring and comparing the stability of proteins expressed from constructs with SECIS elements of four distinct selenoproteins. The estimated Sec incorporation rates are incongruent with the reported SECIS-SBP2 binding equilibrium constants, suggesting the existence of additional factors to explain selenoprotein hierarchy. Our model provides a framework to quantitatively study the regulation of UGA codon redefinition and selenoprotein synthesis.

## Results

### Experimental results

#### Inferring UGA definition using differential protein half-lives between full-length and UGA-terminated selenoproteins

**Fig 1:**
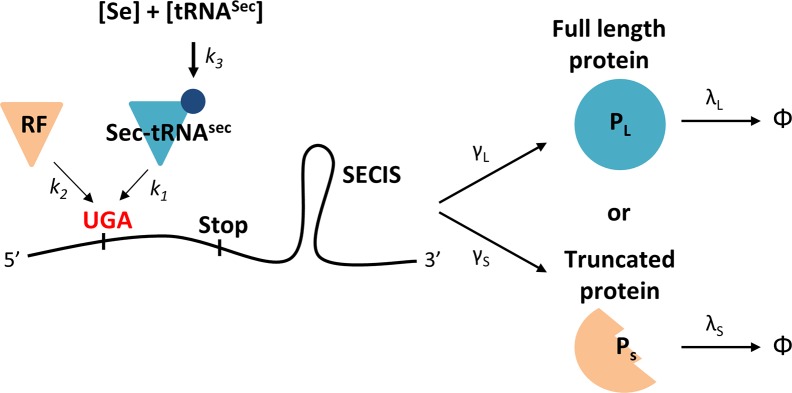
A model of UGA decoding during selenoprotein synthesis. The Sec-tRNA^Sec^ and RFs compete for a UGA site with association constants *k*_1_ and *k*_2_, respectively. The abundance of Sec-tRNA^Sec^ is determined by both selenium (Se) and uncharged tRNA^Sec^ with an equilibrium constant *k*_*3*._ When Sec-tRNA^Sec^ binds to the UGA site, the mRNA will be translated into full length selenoproteins (P_L_) with synthesis rate γ_L_ and degradation rate λ_L_. When RF binds to the UGA site, the mRNA will be translated into truncated proteins (P_S_) with synthesis rate γ_S_ and degradation rate λ_S_. Φ denotes debris after protein degradation.

The UGA codon defines either Sec or translation termination during selenoprotein synthesis. Decoding UGA to Sec or translation termination results in the expression of full-length (P_L_) or truncated (P_S_) selenoproteins, respectively (Fig 1). UGA assignment can therefore be inferred from the ratio of P_L_ to P_S_. We previously found that P_L_ is more stable than P_S_, and that the protein stability of neither species is affected by selenium supply [3]. Thus, our deduction of UGA definition can be taken from the observed protein half-life of the total selenoprotein population (P_T_), which is represented by the mixture of P_L_ and P_S_. Intuitively, when a higher proportion of UGA is redefined as Sec, P_L_ is favored over P_S_, resulting in a greater observed selenoprotein half-life and *vice versa*. This phenomenon can be depicted by the following formula:

$$x\frac{1}{\lambda_{L}}+(1-x)\frac{1}{\lambda_{S}}=\frac{1}{\lambda_{T}}$$

where *x* and (1-*x*) are the proportions of UGA defined as Sec and translation termination, respectively, and *λ*_*L*_, *λ*_*S*_ and *λ*_*T*_ are the degradation rates of P_L_, P_S_ and P_T_, respectively. The reciprocal of *λ* is proportional to the protein half-life. The half-life of P_T_ is a linear combination of that of P_L_ and P_S_, and thus *x* can be deduced by measuring the half-lives of P_T_, P_L_ and P_S_.

#### Characterization of UGA assignments in SEPHS2 and SEPW1 syntheses

We measured the protein half-lives of P_L_, P_S_ and P_T_ using GPS, a single-cell-based dual-fluorescent reporter system [25]. GPS allows for simultaneous measurement of protein synthesis (mRNA level), abundance and half-life using red-fluorescent protein (RFP) signal, green-fluorescent protein (GFP) signal and the GFP/RFP ratio, respectively (S1 Fig). The GPS reporter was permanently integrated into the genome of cells in order to tightly control protein expression levels and to avoid artifacts resulting from transient expression.

**Fig 2:**
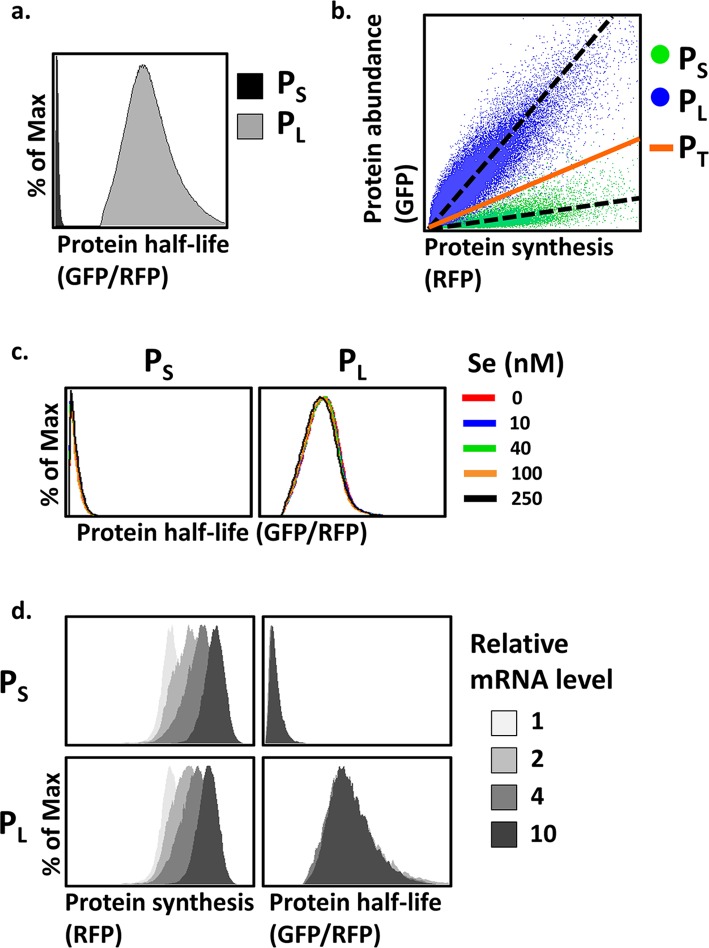
Protein half-life analysis of full-length and truncated SEPHS2. (a) Distributions of protein stability measurements of P_L_ or P_S_ by the GPS assay. P_L_ and P_S_ were expressed from SEPHS2 mutant transcripts that exclusively express one form of SEPHS2. % of Max indicates normalized cell counts such that the peak value of each distribution is 100%. (b) The relationship between protein synthesis and abundance for P_L_ and P_S_. Each dot represents a single cell carrying the indicated GPS reporter with a corresponding protein synthesis (RFP) and protein abundance (GFP). The GFP/RFP ratio, or the slope of the protein synthesis-abundance plot passing through the origin, reflects the protein half-life. A hypothetical line for P_T_, the total amount of proteins of both forms, is shown. (c-d) GPS analysis of P_L_ and P_S_ under various selenium concentrations (c) or synthesis levels (d). Relative mRNA levels are quantifications of the RFP signals in the GPS assay.

We utilized the synthesis of selenoprotein SEPHS2 as a model to investigate the UGA decoding process. To measure the half-lives of P_L_ and P_S_, we generated SEPHS2 mutant transcripts that exclusively express P_L_ or P_S_ (see Experimental Methods for details). Consistent with previous findings [3], P_L_ was much more stable than P_S_ (Fig 2A and 2B), and the half-lives of both species were not affected by either selenium supply (Fig 2C) or protein synthesis rate (Fig 2D). To reiterate the concept of deducing UGA definition using differential protein half-lives between P_L_ and P_S_, a hypothetical curve for wild-type SEPHS2 transcripts (P_T_) expressing both P_L_ and P_S_ is also shown (Fig 2B, the orange line), with the observed half-life lying between P_L_ and P_S_. Favorable UGA to Sec or stop assignments shifts the curve closer to that of P_L_ or P_S_, respectively.

**Fig 3:**
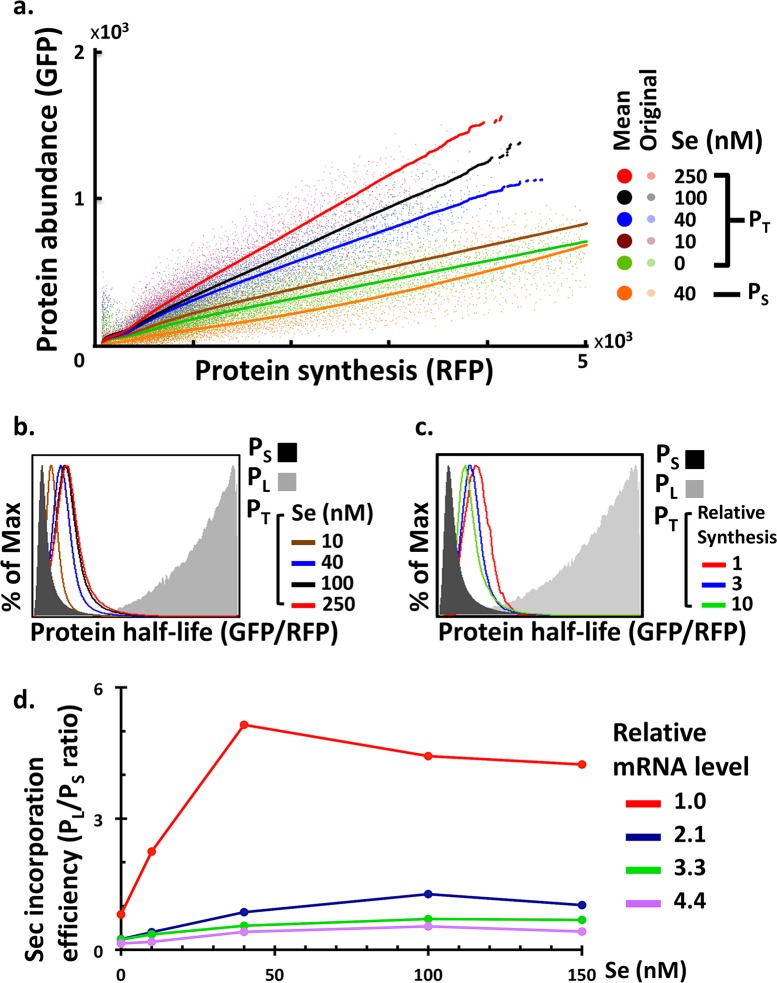
The effect of selenium supply and SEPHS2 synthesis level on UGA definition. (a) The relationship between protein synthesis and abundance for P_T_ analyzed under various selenium concentrations. Both original and processed experimental results are presented in the graph and are represented by “original” and “mean”, respectively. The processed results present the mean abundance at each synthesis level. Since the half-life of P_S_ is not affected by selenium supply, only P_S_ analyzed at 40 nM selenium concentration is shown. (b) GPS assay of P_T_ under various selenium concentrations. (c) GPS assay of P_T_ under 40 nM selenium and various synthesis levels. Relative synthesis levels were estimated from the GPS assay. (d) The ratios of P_L_ and P_S_ abundance were quantified by Western blotting. Relative mRNA levels were estimated from the GPS assay.

To investigate how various factors affected the alternative translation of UGA, we measured the half-lives of P_T_ under different selenium concentrations and synthesis levels. In accordance with the hypothetical line in Fig 2B, the half-lives of P_T_ (i.e. the GFP/RFP ratio or slope) are situated between those of P_L_ and P_S_ (S2 Fig). As revealed by the corresponding increase in the half-lives of P_T_ (Fig 3A and 3B; S2 Fig), UGA to Sec translation is preferred with increasing selenium availability. In contrast, UGA to Sec translation is disfavored with increased SEPHS2 synthesis, as shown by the corresponding decrease in the half-lives of P_T_ (Fig 3C). Consistent with the idea of binding competition between Sec-tRNA^Sec^ and RFs at the UGA sites [26], decreasing the abundance of RFs promoted UGA to Sec decoding as revealed by the increase in the half-life of P_T_ (S3 Fig).

The abundance of P_T_ possesses a positive yet nonlinear relation with SEPHS2 synthesis (Fig 3A). The rate of increase for P_T_ abundance declines with elevated SEPHS2 synthesis. Both the half-life of P_T_ and the amount of protein synthesis that yields a declining rate of P_T_ abundance increase with increasing selenium supply (Fig 3A), suggesting selenium as a limiting factor for UGA to Sec translation. However, elevated selenium supply can only push saturation of P_T_ abundance toward higher protein synthesis but not eradicate it (Fig 3A), and the half-lives of P_T_ cannot reach that of P_L_ even at high selenium concentrations (Fig 3B; S2 Fig). Those observations suggest the existence of additional limiting factors beyond selenium supply.

We directly quantified the protein abundance of P_L_ and P_S_ by Western blotting as an alternative approach to investigate the UGA decoding process. The ratio of P_L_/P_S_ abundance served as an indicator for UGA to Sec translation efficiency (Fig 3D). Consistent with results inferred from protein half-lives (Fig 3A–3C), UGA to Sec translation increased with selenium supply, yet became saturated at high selenium concentrations. The efficiency of UGA to Sec translation declined with synthesis (mRNA levels) at each fixed selenium concentration. Intriguingly, we observed the production of P_S_ even under ample selenium supply (data not shown), suggesting unavoidable binding competition between RFs and Sec-tRNA^Sec^ at the UGA sites.

We analyzed the UGA decoding process of another selenoprotein, SEPW1. The superior stability of the full-length proteins compared to truncated peptides was sustained (S4 Fig). Moreover, the relation between protein abundance and synthesis under various selenium concentrations also possessed similar qualitative characteristics for both SEPW1 (S5A Fig) and SEPHS2 (Fig 3A). In both proteins, protein abundance (GFP) increased with increasing protein synthesis (RFP) and selenium concentrations, yet the GFP-RFP curve slope declined with increasing RFP values. These results indicate that the qualitative behaviors of Sec incorporation are not idiosyncratic to SEPHS2.

#### Comparison of UGA assignments with four SECIS elements

**Fig 4:**
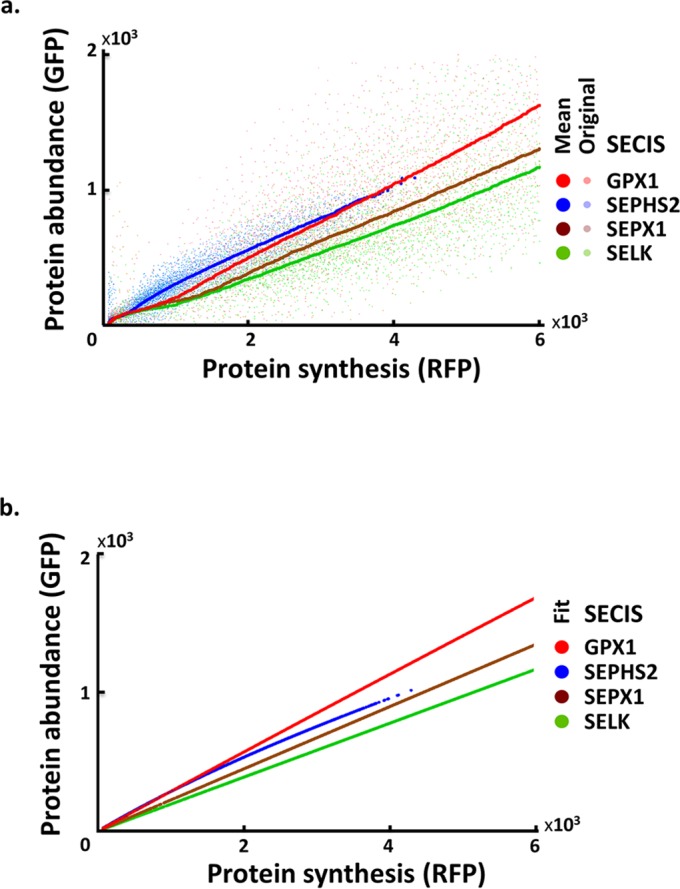
Experimental and predicted Sec incorporation efficiencies in four SECIS constructs. (a) The relationship between protein synthesis and abundance for P_T_ under 40 nM selenium concentration in the experimental data of four SECIS constructs: GPX1 (red), SEPHS2 (blue), SEPX1 (brown), and SELK (green). A solid circle indicates the mean GFP value for each RFP value. (b) The relationship between protein synthesis and abundance for P_T_ under 40 nM selenium concentration according to the models inferred from experimental data.

To investigate the role of SECIS elements on hierarchical selenoprotein translation, we replaced the SECIS element of the SEPHS2 transcript with those from three other selenoprotein transcripts–GPX1, SELK, and SEPX1 –and monitored SEPHS2 protein expressions by the GPS assay. We show the relations between protein abundance and protein synthesis under 40 nM selenium concentration (Fig 4A) and four selenium concentrations (S6A–S6D Fig). The data from those constructs exhibited a hierarchy of Sec incorporation efficiency. SEPHS2 and GPX1 had higher GFP-RFP slopes than SEPX1 and SELK, indicating superior Sec incorporation of the SECIS elements of SEPHS2 and GPX1 to those of SEPX1 and SELK.

### Computational modeling results

#### A mathematical model of the Sec incorporation process

We propose a simple mechanistic model of selenoprotein expression control that accounts for the aforementioned experimental characteristics:

1. P_L_ is more stable than P_S_, and their half-lives are not affected by synthesis level or selenium supply.
2. Total selenoprotein abundance increases with both mRNA levels and selenium supply.
3. Additional limiting factors account for the saturation of P_T_ at high levels of selenoprotein mRNA and selenium.
4. UGA to Sec translation increases with selenium supply but decreases with selenoprotein mRNA levels, and it saturates at high selenium concentrations due to the existence of the same limiting factor.
5. Constituent binding competition between RFs and Sec-tRNA^sec^ occurs at UGA sites.

The model is schematically illustrated in Fig 1 and described below.

#### Basic reactions and hypotheses

The model is based on the following simplifying assumptions:

1. Synthesis and degradation reactions of both P_L_ and P_S_ follow first-order kinetics, which stipulate that the reaction rates are proportional to the substrate concentrations.
2. P_L_ and P_S_ have distinct synthesis and degradation rates. P_S_ possess a considerably shorter half-life than P_L_.
3. RFs and Sec-tRNA^Sec^ compete for UGA sites.
4. The total amount of selenoprotein mRNA is distributed among the transcripts participating in the translation of P_L_ (mRNA-Sec-tRNA^Sec^), P_S_ (mRNA-RF), and free molecules.
5. The total amount of tRNA^Sec^ in a cell is fixed and distributed between free and charged tRNAs.
6. The conjugation of Sec to tRNA^Sec^ also follows first-order kinetics with respect to selenium and free tRNA^Sec^ molecules.

The selenoprotein constructs in our experiments are derived from intron-less cDNAs and thus are immune to nonsense-mediated mRNA decay (NMD), a well-known mRNA quality surveillance mechanism to eliminate mRNA with premature stop codons [27, 28]. Nevertheless, we have incorporated NMD regulation into our model (Eqs 5–7 in Materials and Methods).

Under those assumptions, we describe the following reactions at steady state in this model:

1. Sec-tRNA^Sec^ incorporation, P_L_ translation and degradation:
  $$[mRNA]+[{Sec-tRNA}^{Sec}]\;\overset{k_{1}}{↔}\;[{mRNA-Sec-tRNA}^{Sec}]\;\overset{\gamma_{L}}{\to }\;[P_{L}]\;\overset{\lambda_{L}}{\to }\;\emptyset$$
2. RF competition for the UGA site, P_S_ translation and degradation:
  $$[mRNA]+[RF]\overset{k_{2}}{↔}[mRNA-RF]\;\overset{\gamma_{S}}{\to }\;[P_{S}]\;\overset{\lambda_{S}}{\to }\;\emptyset$$
3. Sec-tRNA^Sec^ synthesis:
  $$\left[Se]+[{{tRNA}^{Sec}}_{free}]\overset{k_{3}}{↔}[{{Sec-tRNA}^{Sec}}_{total}\right]$$

In addition to the aforementioned reactions, we also imposed three other constraints on the total amounts of selenoprotein mRNA and tRNA^Sec^. The first constraint stipulates that the selenoprotein mRNAs are distributed among the molecules bound to Sec-tRNA^Sec^, RFs, and free molecules (Eq 10). The second constraint stipulates that the total Sec-tRNA^Sec^ molecules are distributed between the charged tRNAs interacting with mRNAs and free molecules (Eq 11). The third constraint stipulates that the total tRNA^Sec^ molecules are distributed between charged and uncharged tRNAs (Eq 12).

The model consists of nine parameters: translation (γ) and degradation (λ) rates of P_L_ and P_S_ (i.e. γ_L_/λ_L_ and γ_S_/λ_S_ respectively); equilibrium constants for the interactions with Sec-tRNA^Sec^ and RFs (*k*_*1*_ and *k*_*2*_, respectively); the equilibrium constant of charging Sec to tRNA^Sec^ (*k*_*3*_); the total amount of tRNA^Sec^ (*T*_*total*_); and the total amount of RFs. The total amounts of mRNA and protein levels (*m*_*total*_ and *P*_*total*_ respectively) of each cell are measured by the RFP and GFP intensities in the GPS assay (Eq 13). Given those parameters as well as the equations and constraints derived from the hypotheses above, the relationship between total protein abundance and total mRNA levels can be expressed as a complex functional formula.

To estimate the model parameters, we further simplified the nine parameters in the model and combined them into six independent parameters: the ratios of synthesis and degradation rates γ_L_/λ_L_ and γ_S_/λ_S_ were calculated from the experimental data of P_L_ and P_S_ alone, respectively; *k*_*2*_ and *RF* were combined into one parameter *kF* as they always co-occurred in the equations; we also introduced parameters ρ_p_ and ρ_m_ to specify the ratios of protein and mRNA abundance from GFP and RFP intensities, respectively, and replaced ρ_m_ with an equivalent parameter ρ = ρ_m_/ρ_p_. Consequently, only the following six parameters need to be estimated: *k*_*1*_, *kF*, *k*_*3*_, *T*_*total*,_ ρ and ρ_p._ A detailed description of the model is reported in Materials and Methods.

#### Recapitulation of the qualitative characteristics of selenoprotein synthesis and degradation

To verify the sensibility of this model, we examined if it could reproduce the qualitative properties observed from experimental data for SEPHS2. Moreover, to ensure that this model consists of all the essential requirements to explain the observed phenomena, we excluded the two constraints (mRNA and tRNA^Sec^), both separately and together, and checked whether the reduced models could still recapitulate the same qualitative properties.

**Fig 5:**
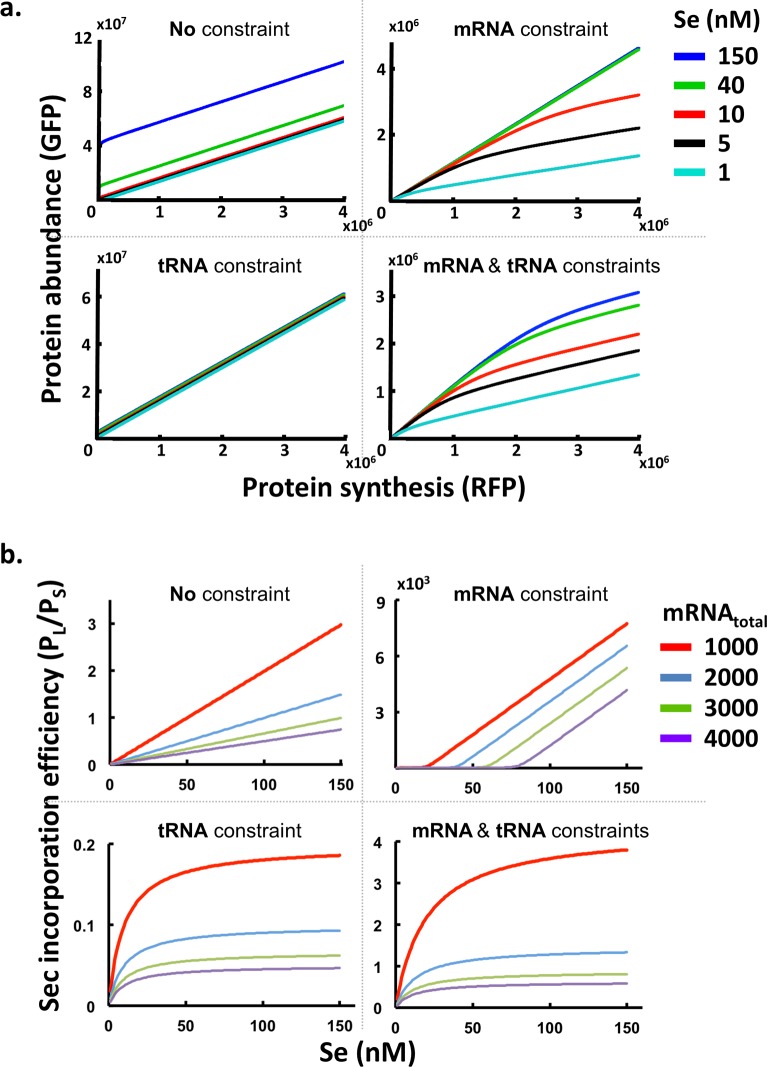
Prediction of Sec incorporation efficiencies under four different constraints. (a) Protein abundance among various protein synthesis rates and selenium concentrations was simulated using mathematical models. Four models were compared using a specific parameter set ({*k*_1_, *k*_3_, *k*_*f*_, *T*_*total*_, *ρ*, *ρ*_*p*_} = {3, 10, 0.1, 500, 10, 100}; [*m*_*total*_] = 1∼4000). (b) Simulation of Sec incorporation efficiency (P_L_/P_S_ ratio) using parameter sets identical to (a). The caption “mRNA total” indicates the number of mRNA molecules in the model.

We selected a specific set of parameter values in the model ({*k*_1_, *k*_3_, *k*_*f*_, *T*_*total*_, *ρ*, *ρ*_*p*_} = {3, 10, 0.1, 500, 10, 100}; [*m*_*total*_] = 1∼4000), varied the amount of selenium supply and mRNA levels, and then generated simulated data for the GPS assay (Fig 5A) and the Western blot experiment (Fig 5B). We compared the simulation outcomes of four models: (1) the model with both mRNA and tRNA^Sec^ constraints; (2) the model with the mRNA constraint alone; (3) the model with the tRNA^Sec^ constraint alone; and (4) the model without mRNA and tRNA^Sec^ constraints. Only the model incorporating both constraints exhibits saturation of the total protein abundance (Fig 5A) and P_L_/P_S_ (Fig 5B) with increased protein synthesis and selenium supply, respectively. At low mRNA (protein synthesis) levels, P_L_ formation dominates due to its superior stability. Hence, observed protein stability is higher, as indicated by the slope of the protein abundance-synthesis curve (Fig 5A, lower-right panel, left part of the curves) and the higher P_L_/P_S_ (Fig 5B, lower-right panel, the red curve). As the mRNA level increases, Sec-tRNA^Sec^ molecule supply becomes exhausted and P_S_ formation dominates. Therefore, the observed protein stability approaches the lower rate of P_S_ (Fig 5A, lower-right panel, right part of the curves), and P_L_/P_S_ becomes smaller (Fig 5B, lower-right panel, the purple curve). Similarly, at low selenium concentrations, there is an abundant supply of uncharged tRNA^Sec^. Thus, the amount of charged Sec-tRNA^Sec^ is proportional to the selenium concentration, and the amount of P_L_ produced is roughly proportional to Sec-tRNA^Sec^ supply (Fig 5B, lower-right panel, left part of the curves). When selenium concentration increases, all tRNA^Sec^ molecules are charged. Thus, P_L_ formation depends only on the amount of tRNA^Sec^ and becomes insensitive to selenium concentration (Fig 5B, lower-right panel, right part of the curves). Increasing mRNA levels enhance incorporation of Sec and depletion of uncharged tRNA^Sec^ molecules, thereby pushing saturation of the P_L_/P_S_ ratio towards lower selenium concentrations (Fig 5B, lower-right panel).

Both the mRNA and tRNA^Sec^ constraints are essential to reproduce the qualitative characteristics observed from experimental data. The model with only the mRNA constraint can account for the lower translational efficiency at higher mRNA levels due to the dominance of P_S_ (Fig 5A, upper-right panel), in accordance with our experimental results from GPS assay (Fig 3A). However, since the tRNA^Sec^ supply is unlimited, the charged Sec-tRNA^Sec^ abundance is proportional to the selenium concentration. P_L_ formation is therefore linearly dependent on the selenium concentration when it is high (Fig 5B, upper-right panel), which cannot explain the results from our Western blot experiment (Fig 3D). The intervals with zero P_L_/P_S_ reflect the regimes where charged Sec-tRNA^Sec^ become a limiting factor. In contrast, the model with only the tRNA^Sec^ constraint can recapitulate the saturation of P_L_ formation at high selenium concentrations due to limited tRNA^Sec^ supply (Fig 5B, lower-left panel), in accordance with the results from our Western blot experiment (Fig 3D). However, since free mRNA supply is unconstrained, the maximum capacity to produce P_L_ is quickly reached (due to limited tRNA^Sec^ supply), and formation of P_S_ dominates subsequent protein synthesis. Thus, the protein abundance-synthesis curves are straight and are collapsed into a single line for all selenium concentrations (Fig 5A, lower-left panel), which cannot explain the experimental results from our GPS assay (Fig 3A). The model without either constraint does not exhibit non-linearity in either experiment (Fig 5A and 5B, upper-left panels).

#### Estimation of model parameters

The six independent parameters were connected by complex nonlinear functional relationships. We developed an algorithm to estimate the parameters that fit the functional relationships between single-cell GFP and RFP intensities from our GPS experiments. In brief, each set of parameters *π* gave rise to a function *GFP* = *f*_*π*_(*RFP*). We defined the loss function as the square error between measured and predicted GFP values, summing over all data points: *Q*^2^(*π*) = ∑_*i*_(*GFP*_*i*_ − *f*_*π*_(*RFP*_*i*_))^2^. A grid-search algorithm was employed to find the parameter values that minimized the loss function. The procedures for data processing and parameter estimation are described in Materials and Methods.

#### The parameter estimation algorithm can recover parameter values from simulations

To see how precisely our algorithm recovered the parameter values, we performed a simulation test. We generated 100 random parameter combinations (Eq 18) and simulated the corresponding RFP versus GFP data points for each parameter set. The algorithm estimated the parameter values based on simulated data points. By comparing the input and predicted parameters, we evaluated the success rate of recovering correct parameters (see Material and Methods). The success rate varied between 70–100% with the highest resolution of grid search (Table 1, the last row). The average recovery rate ranged from 64% to 76% with grid densities increasing from 1024 (4 possible values for each parameter) to 248832 (12 possible values for each parameter) within the same parameter boundary. We also introduced noise in simulated data points and assessed the parameter recovery rates from noisy data (see Material and Methods, Eq 19). Experimental data indicated that the noise of GFP values for a given RFP value is proportional to the RFP signal level, and the standard deviation of the normalized noise is about 0.3. We varied standard deviation of the noise in simulated data from 0.3 to 5 and report the recovery rate in Table 2 (see Materials and Methods). The recovery rate varied from 70% to 33% as the normalized standard deviation of noise increased from 0 to 5. The recovery rate dropped below 50% when the normalized noise standard deviation is above 1.0. These results are intuitive, as it is hard to reconstruct a model when noise exceeds the signal level.

**Table 1:** Parameter recovery rate under different resolutions.

	Parameters					
Blocks	*k*_*1*_	*kF*	*k*_*3*_	*T*_*total*_	*ρ*	Average
4	61%	58%	100%	68%	69%	64%
6	63%	63%	100%	73%	80%	70%
8	67%	68%	100%	69%	77%	70%
10	68%	73%	100%	77%	79%	74%
12	76%	71%	100%	76%	80%	76%

The table shows the recovery rate of each parameter from 100 simulations. A successful recovery is defined when the recovered parameter values are within ten-fold of the true parameter values. It shows the recovery rate under different algorithm resolutions. The “Blocks” column indicates the grid numbers used in the algorithm. Higher grids result in higher resolution.

**Table 2:** Parameter recovery rate under different levels of data noise.

Parameters						
Data std.	*k*_*1*_	*kF*	*k*_*3*_	*T*_*total*_	*Ρ*	Average
0.0	67%	68%	100%	69%	77%	70%
0.3	53%	50%	77%	64%	54%	55%
0.6	43%	45%	64%	55%	48%	48%
1.0	48%	55%	71%	56%	49%	52%
3.0	39%	38%	61%	44%	42%	41%
5.0	28%	32%	51%	39%	33%	33%

The table shows the recovery rate under different data noise levels. The column “Data Std.” indicates the standard deviations of the noisy data sets (see Material and Methods).

#### Estimated parameter values from GPS data

We employed the grid search algorithm to estimate the six independent parameters from the SEPHS2 GPS data. Table 3 displays the top 10 parameter sets identified by the algorithm. They are grouped into two degenerate classes of solutions. Within each class, each parameter set gives rise to the same loss function value. Among them, the highest loss function value is 2.1-fold that of the lowest one. The differences between respective *k*_*3*_, *T*_*total*_ and ρ_p_ values are all within 1.5-fold. Greater differences between minimum and maximum values occurred for *k*_*1*_ (1.4-fold) and ρ_p_ (1.5-fold). Small differences between the top-ranking parameter values obtained from a global grid search suggest their closeness to the global optimum values.

**Table 3:** Top ten estimated SEPHS2 parameters from experimental data.

*k*_*1*_	*kF*	*k*_*3*_	*T*_*total*_	*ρ*	*ρ*_*p*_	*Q*^*2*^
17.03	9803.03	0.02	63.44	10.40	0.95	1.48 x 10^8^
17.65	9704.55	0.02	60.63	10.87	0.95	1.48 x 10^8^
17.96	10000.00	0.02	61.39	10.75	0.95	1.48 x 10^8^
17.03	9556.83	0.02	61.90	10.64	0.95	1.48 x 10^8^
17.34	9852.28	0.02	62.67	10.52	0.95	1.48 x 10^8^
13.13	8621.24	0.02	72.39	13.32	0.65	3.17 x 10^8^
12.82	8473.52	0.02	72.90	13.20	0.65	3.17 x 10^8^
13.13	9768.97	0.02	73.66	13.09	0.65	3.17 x 10^8^
13.13	8867.45	0.02	74.43	12.97	0.65	3.17 x 10^8^
12.82	8621.24	0.02	74.18	12.97	0.65	3.17 x 10^8^

**Fig 6:**
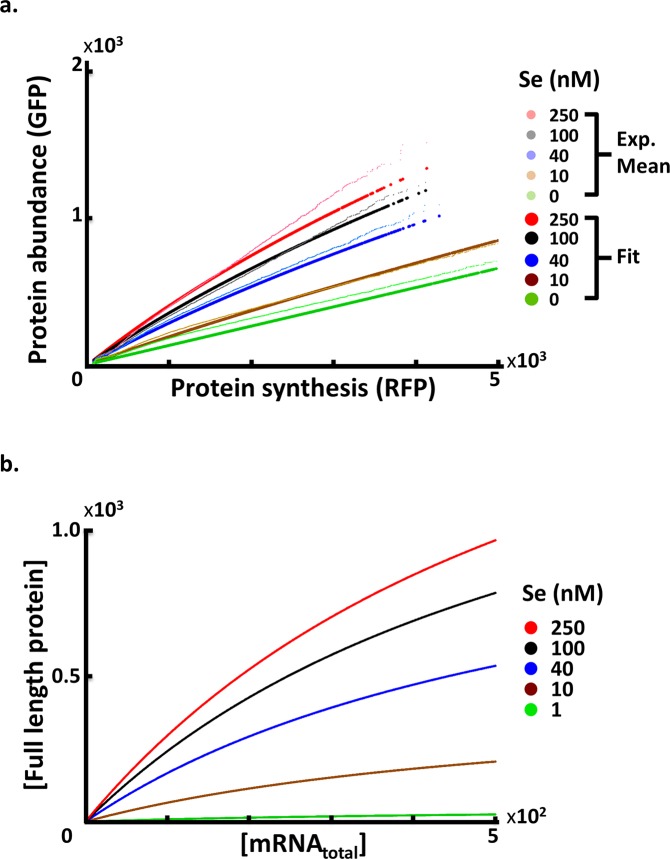
Comparison of experimental and predicted Sec incorporation efficiencies in SEPHS2. **(a)** The relationship between protein synthesis and abundance for P_T_ analyzed under various selenium concentrations. Dots denote mean GFP values for each RFP value in the experimental data and are the same as Fig 3A. Solid circles denote the same quantities from model fitting. (b) The relationship between the full length protein quantities and mRNA levels under various selenium concentrations from model prediction.

We checked how well the model derived from the top-ranking parameter values fit the experimental data. Since the scattered plots of GFP-RFP intensities of the GPS data were noisy, we show the mean of GFP values corresponding to each single RFP value (Fig 6A). The GFP-RFP curves generated by the optimum parameter values (solid circles) fit well with the experimental data (dots) at high selenium concentrations (red, black and blue colors). At the lowest selenium concentration, the model underestimates the GFP value (protein abundance) with each fixed RFP value (mRNA level) (green dots and circles). This shift is likely due to the existence of endogenous selenium in cells with little or no external selenium supply. Beyond qualitative observations in Figs 3A, 3D and 5, we also compared two quantitative scores of goodness of fit (*r*^*2*^ and root mean square error, RMSE) among three alternative models (with mRNA and tRNA constraints alone and a combination of both constraints) of the data from GPS (S1 Table) and Western blot (S2 Table) assays.

We also checked whether the estimated parameter values were within biologically sensible ranges according to prior studies (Table 4). In mammalian cells, the ratios of protein synthesis and degradation rates have a broad spectrum of values, ranging from 10^−3^ to 10^4^ [29]. The SEPHS2 protein synthesis/degradation ratio calculated from our control experiments varies from 70 to 80, which falls within this range. We also estimated the possible ranges of mRNA and protein copy numbers of SEPHS2. Previous studies have reported an SEPHS2 mRNA expression level of approximately 10^2^ molecules per cell and a protein expression level of 10^3^ molecules per cell [29–31] (see Material and Methods). The mRNA and protein levels in our results are all within these ranges (Fig 6B).

**Table 4:** Physiological ranges of the model parameters.

Parameter	Description	Typical values	References
***k***_***1***_	mRNA-Sec-tRNA^Sec^ association constant	0~100 (nM/hr)	[29, 35]
***kF***	Product of release factor concentration and mRNA-release factor association constant (*k*_*2*_)	0~10000	[31]
***k***_***3***_	Sec-tRNA^Sec^ synthesis rate	0~100 (nM/hr)	[36]
***T***_***total***_	Abundance of tRNA^Sec^ in the cell	10~1000 (nM)	[37–40]
***ρ***	Ratio of RFP and GFP intensity constant	0~10000	[29, 41]
***ρ***_***p***_	GFP intensity constant	0~10000 (a.u./nM)	[29, 41]
***s1***	Ratio of γ_L_ over λ_L_	Estimated from FACS data	[42]
***s2***	Ratio of γ_S_ over λ_S_	Estimated from FACS data	[42]

To justify the wider applicability of the model estimation algorithm, we estimated the model parameters of SEPW1 from the experimental data (S3 Table). Similar to SEPHS2, the GFP-RFP curves of SEPW1 generated by the inferred model (S5B Fig) recapitulates the qualitative characteristics of experimental data (S5A Fig).

#### Comparison of Sec incorporation rates and SECIS-SBP2 binding affinity in selenoproteins

We replaced the SECIS element of the SEPHS2 transcript with those from three other selenoprotein transcripts to investigate the role of SECIS elements on hierarchical selenoprotein expression. We estimated the model parameters of the four SECIS constructs, compared their *k*_*1*_ and *kF* values in Table 5, and reported all the inferred parameter values in S4 Table. While all the models possess a similar level of *kF*, their *k*_*1*_s can be separated into two groups: SEPHS2 and GPX1 have higher values (17.0 and 12.2) than SEPX1 and SELK (5.7 and 5.7) (Fig 4B). This order is compatible with the order of GFP-RFP curves in experimental data (Fig 4A and S6 Fig). Similar levels of *kF* are consistent with the experimental setting, as all the constructs are derived from SEPHS2 and differ only in their SECIS elements. Their RF incorporation efficiency (*k*_*2*_) and RF concentration should thus be invariant. Likewise, other parameters pertaining to the processing of alternative UGA codon assignments (*k*_*3*_ and *T*_*total*_) also exhibit similar levels (S4 Table).

**Table 5:** Comparison of estimated Sec incorporation strength of four SECIS elements and SECIS-SBP2 dissociation constants.

SECIS element	SECIS-SBP2 *K*_*d*_ (nM)	*k*_*1*_	*kF*	*k*_*1*_/ *kF*
SEPHS2	7.5	17.0	9803.0	0.0017
GPX1	6.3	12.2	8521.3	0.0014
SEPX1	9.7	5.7	9803.0	0.0006
SELK	3.6	5.7	8522.8	0.0007

The values of *k*_*1*_ and *kF* for each SECIS element were estimated from their experimental data. The SECIS-SBP2 disassociation constants *K*_*d*_ are reported from [43].

However, the order of Sec incorporation efficiency among the four SECIS elements (*k*_*1*_) is not compatible with their SECIS-SBP2 binding disassociation constants (*K*_*d*_ in Table 5). In particular, SELK possesses the lowest disassociation constant (thus the highest SECIS-SBP2 binding affinity), yet has the lowest Sec incorporation efficiency. The order of SECIS-SBP2 binding affinity among the remaining three SECIS elements (GPX1, SEPHS2, SEPX1) is roughly compatible with the order of their *k*_*1*_ values (SEPHS2, GPX1, SEPX1).

## Discussion

Selenoprotein synthesis serves as a remarkable model to study how cellular and environmental factors influence the definition of a dual-use codon. We have proposed a concise mathematical model of selenoprotein synthesis that matches well with both qualitative and quantitative characteristics of experimental results. By combining the power of biological experiments and computational modeling, we have revealed how multiple *cis* and *trans* regulatory factors collectively influence the definition of UGA.

The characteristics of experimental data can be explained by the competition between RF and Sec-tRNA^Sec^ for UGA codons of limited selenoprotein mRNAs, as well as the limited abundance of tRNA^Sec^. We formulated these two types of resource limitation as a quantitative, mechanistic model. Simulations according to this model successfully reproduced qualitative characteristics of the experimental data (Fig 5). Beyond qualitative matching, we also proposed an algorithm to estimate model parameters from experimental data. The model derived from the estimated parameters fit well with the experimental data (Fig 6A, S1 Table and S2 Table).

Previous work on the importance of SECIS-SBP2 interactions for the selenoprotein expression hierarchy remains inconclusive. Some studies have indicated that SECIS-SBP2 interactions dictate the selenoprotein hierarchy [8], whereas others have suggested that those interactions alone are insufficient to determine Sec incorporation efficiency [21, 32]. Our deduced Sec incorporation rates attributed to distinct SECIS elements did not correlate well with reported SECIS-SBP2 binding affinities (Table 4). SEPHS2 and GPX1 had substantially higher Sec incorporation rates than SEPX1 and SELK, yet the SECIS-SBP2 binding of SELK was the strongest among the four SECIS elements. Thus, we provide evidence to support the presence of other determining factors for selenoprotein hierarchy.

The order of predicted GFP-RFP curves among the four SECIS elements is consistent with the order of the corresponding experimental curves except for zero selenium concentration (S6 Fig). At zero selenium concentration, the predicted curves of all SECIS elements coincide and are considerably lower than all the experimental curves. This is likely due to the existence of residual selenium in cells even at zero external selenium supply.

The parameters in our model conform to some of the fundamental quantitative features of cell biology, such as the translation and degradation rates of proteins, incorporation rates of Sec-tRNA^Sec^ and RFs, and the quantities of tRNA^Sec^ and RFs in cells. Few of these quantities have been reported for mammalian cells, so it is not possible to verify the accuracy of the estimated parameters from existing information. Thus, a thorough verification of the estimated parameter values remains to be conducted.

The concise selenoprotein synthesis model we propose circumvents detailed mechanistic description. It is now possible to build a more detailed, mechanistic model by including all the intermediate steps in the pathway. However, introducing additional free parameters without concomitant measurements merely complicates the model with little improvement in accuracy. Importantly, in our simplified equations, we reveal the existence of a limiting factor beyond selenium concentration in Sec-tRNA^Sec^ synthesis. Which enzymes or substrates constitute the true limiting factor warrants further investigation. Likewise, incorporation of tRNA^Sec^ or RFs at a UGA site involves binding of multiple molecules [8–12, 33, 34]. Some of them could possibly be limiting factors additional to excess mRNA and tRNA^Sec^ supplies.

Despite Sec incorporation being a very specialized process, the process of synthesizing and degrading multiple products with shared and limited resources is ubiquitous in biochemical systems. Some instances include dichotomy between growth and production of organisms, competitive binding of transcription factors and their repressors on promoters, and biosynthesis of metabolites from multiple pathways with shared substrates. Although the models capturing those phenomena may have very different formulations than the models described in this study, the methodology we introduced may be extended to other systems with similar characteristics. Furthermore, presence of multiple exogenous and endogenous limiting factors, such as selenium, selenoprotein transcripts and tRNA^Sec^ in our study, may yield a more complicated system behavior than the cases with single or no limiting factors.

## Materials and methods

### Plasmid construction

To generate the SEPHS2 and SEPW1 GPS reporter construct, SEPHS2 and SEPW1 cDNA from the Mammalian Gene Collection (GE Healthcare Dharmacon Inc., Lafayette, CO, USA) was cloned into a lentiviral vector carrying the RFP-IRES-GFP GPS cassette using Gateway technology (Life Technologies, Carlsbad, CA, USA). To generate SEPHS2 and SEPW1 mutants that exclusively express P_L_ or P_S_, the TGA/Sec codon on SEPHS2 and SEPW1 cDNA was mutated into TGT/Cys or TAA/stop by site-directed mutagenesis (Stratagene, Santa Clara, CA, USA), respectively.

To replace the SECIS element of SEPHS2 with that of other selenoproteins, SECIS elements of GPX1, SELK and SEPX1 were amplified from corresponding selenoprotein cDNAs and cloned into the SEPHS2 reporter using Gibson Assembly (New England Biolabs Inc., Ipswich, MA, USA).

### Tissue culture

HEK293T cells were maintained in DMEM with 10% fetal bovine serum (FBS, purchased from Hyclone Laboratories, Logan, UT, USA) and antibiotics in a 6% CO_2_ atmosphere at 37°C. FBS is the main source of selenium in cell culture. To control selenium supply, cells were first depleted of selenium in FBS-free DMEM supplemented with 10 μg/mL insulin and 5 μg/mL transferrin for 24 hrs. Cells were then balanced with indicated concentrations of sodium selenite (Na_2_SeO_3_, Sigma-Aldrich, St. Louis, MO, USA) for another 24 hrs. All tissue culture media and supplements were purchased from Gibco Life Technologies, unless otherwise indicated.

To produce lentiviruses, HEK293T cells were transfected with pHAGE, pHIV gag/pol, pVsvg, pRev and pTat using TransIT-293 reagent (Mirus Bio LLC, Madison, WI, USA). Viruses were harvested 48 hrs after transfection.

### Generation of GPS reporter cell lines and GPS assays

To generate GPS reporter cell lines, cells were infected with lentiviruses carrying GPS reporter constructs. Infection was carried out in media with 8 μg/mL polybrene (Sigma-Aldrich). To collect reporter cell lines with a series of SEPHS2 synthesis levels, cells were infected stepwise with lentiviruses carrying GPS reporter constructs. To prepare samples for FACS analysis, cells were washed with PBS, trypsinized and resuspended in medium containing 2% FBS and analyzed using a BD LSR Fortessa system (BD Biosciences, San Jose, CA, USA). 10^6^ cells were recorded for each sample. FlowJo (Ashland, OR, USA) was used for primary FACS data analysis.

### Western blotting

Cells were harvested in cold PBS and lysed in RIPA buffer (150 mM NaCl, 1.0% IGEPAL®CA-630, 0.5% sodium deoxycholate, 0.1% SDS, and 50 mM Tris, pH 8.0). Standard procedures were used for Western blotting. Antibody against GFP (JL-8) was purchased from Clontech Laboratories (Mountain View, CA, USA).

### Data processing

The single-cell-based GPS data consists of 10^6^ pairs of RFP-GFP intensities for individual cells. The RFP-GFP relationship in each cell manifests a high level of variation. However, for each small range of RFP values, the corresponding GFP values typically have a Gaussian distribution with a variance proportional to the RFP value. Therefore, we treated the GPS data as instantiations of the following random variables: *y* = *f*(*x*) + *ϵ*, where *x* denotes a random variable of RFP intensities with an unspecified distribution and *y* denotes a random variable of GFP intensities and is a function of *x* with an additive noise *ϵ*. *ϵ*∼*N*(0,*xσ*^2^) follows a Gaussian distribution with zero mean and *xσ*^2^ variance.

To reduce data noise and size, we applied two filtering procedures to the GPS data. First, we divided the range of RFP and GFP values into 2000 grids and discarded the data points in grids comprising fewer than 30 data points. Second, we sorted the RFP values and selected 0.4% data points. The processed data thereby consisted of about 3000 pairs of RFP and GFP values for each selenium concentration.

### A mathematical model of selenoprotein synthesis and degradation

The basic assumptions and reactions of the model are described in the Results and illustrated in Fig 1. Here, we demonstrate the mathematical formulation of the model. We first introduce the following notations:

- *m*_*total*_: concentration of total selenoprotein mRNA molecules
- *m*_*f*_: concentration of free selenoprotein mRNA molecules not interacting with Sec-tRNA^Sec^ or RFs.
- *SeT*_*f*_: concentration of free Sec-tRNA^Sec^ molecules
- *m* − *SeT*_0_: concentration of the mRNA-Sec-tRNA^Sec^ complex before mRNA degradation
- *m* − *SeT*: concentration of the mRNA-Sec-tRNA^Sec^ complex after mRNA degradation
- *k*_1_: association constant of the reaction *m*_*f*_ + *SeT*_*f*_ ⇌ *m* − *SeT*
- *P*_*L*_: concentration of full-length selenoproteins
- *γ*_*L*_: translation rate of full-length selenoproteins
- *λ*_*L*_: degradation rate of full-length selenoproteins
- *RF*: concentration of RFs
- *m* − *RF*_0_: concentration of the mRNA-RF complex before mRNA degradation
- *m* − *RF*: concentration of the mRNA-RF complex after mRNA degradation
- *k*_2_: association constant of the reaction *m*_*f*_ + *RF* ⇌ *m* − *RF*
- *P*_*S*_: concentration of truncated selenoproteins
- *γ*_*S*_: translation rate of truncated selenoproteins
- *λ*_*S*_: degradation rate of truncated selenoproteins
- *Se*: selenium concentration
- *T*: concentration of uncharged tRNA^Sec^
- *SeT*_*total*_: concentration of charged Sec-tRNA^Sec^
- *T*_*total*_: concentration of all tRNA^Sec^ molecules (charged and uncharged combined)
- *k*_3_: association constant of the reaction *T* + *Se* ⇌ *SeT*_*total*_
- *α*_*L*_: probability that an mRNA-Sec-tRNA^Sec^ complex escapes mRNA degradation
- *α*_*S*_: probability that an mRNA-RF complex escapes mRNA degradation
- *e*_0_: background mRNA decay rate
- *N*: average number of proteins translated from one mRNA molecule during its life

#### Full-length protein synthesis and degradation

At equilibrium, *m* − *SeT*_0_ is proportional to the product of *m*_*f*_ and *SeT*_*f*_ prior to mRNA degradation:

$$k_{1}\cdot m_{f}\cdot SeT_{f}=m-SeT_{0}$$ (1)

A fraction of *m* − *SeT*_0_ complexes are degraded by the background mRNA decay process.

$$m-SeT=\alpha_{L}\cdot m-SeT_{0}$$ (2)

$$\alpha_{L}=1-e_{0}$$ (3)

Likewise, at steady state, the total amounts of translated and degraded molecules are equal:

$$m-SeT\cdot \gamma_{L}=P_{L}\cdot \lambda_{L}$$ (4)

#### Truncated protein synthesis and degradation

The equations for truncated protein synthesis and degradation follow those of full-length proteins by replacing Sec-tRNA^Sec^ with RFs:

$$k_{2}\cdot m_{f}\cdot RF=m-RF_{0}$$ (5)

$$m-RF=\alpha_{S}\cdot m-RF_{0}$$ (6)

$$\alpha_{S}=1-\frac{1}{\left[\left(N-1\right)\eta \right]}-\frac{\left[e_{0}\left(N-1\right)\eta \right]}{\left[\left(N-1\right)\eta +1\right]}$$ (7)

$$m-RF\cdot \gamma_{S}=P_{S}\cdot \lambda_{S}$$ (8)

where $\eta =\frac{k_{1}∙SeT_{f}}{k_{1}∙SeT_{f}+k_{2}∙RF}$ is attributed to NMD. Derivation of *α*_*L*_ and *α*_*S*_ is described in S1 File. Since mRNA degradation can be neglected in our system, we set *α*_*L*_ = *α*_*S*_ = 1.

#### Sec-tRNA^Sec^ synthesis

We simplified the complicated process of Sec-tRNA^Sec^ synthesis to a first-order reaction that depends bilinearly on selenium concentration and uncharged tRNA^Sec^:

$$SeT_{total}=k_{3}\cdot T\cdot Se$$ (9)

#### mRNA constraint

The mRNA constraint simply states that the selenoprotein mRNAs are allocated among the mRNA-Sec-tRNA^Sec^ complexes, mRNA-RF complexes, and free mRNAs:

$$m_{f}+m-SeT+m-RF=m_{total}$$ (10)

#### tRNA constraints

There are two constraints involving tRNA^Sec^. First, the total amount of charged tRNA^Sec^ is distributed between the Sec-tRNA^Sec^ molecules interacting with mRNAs and the free Sec-tRNA^Sec^ molecules:

$$SeT_{f}+m-SeT=SeT_{total}$$ (11)

Second, the total amount of tRNA^Sec^ is distributed between charged and uncharged species:

$$T+SeT_{total}=T_{total}$$ (12)

#### Conversion of fluorescence intensities into mRNA and protein abundance

The GPS assay measures fluorescence intensities rather than molecular abundance. To convert the RFP and GFP intensities into mRNA and protein abundance, we introduced two additional parameters:

$$P_{total}=\frac{GFP}{\rho_{\text{p}}},\;m_{total}=\frac{RFP}{\rho \cdot \rho_{\text{p}}}$$ (13)

#### Reduction of model parameters

The number of parameters appearing in Eqs 1–8 can be reduced in the following way. First, we collapsed *k*_2_ ∙ *RF* into a single parameter *kF* as they always co-occurred in the equations. Second, only the translation/degradation rate ratios *γ*_*L*_/*λ*_*L*_ and *γ*_*S*_/*λ*_*S*_ are relevant in our experiments. Third, those ratios can be directly determined from the control experiments with complete full-length or truncated protein synthesis (Fig 2B): $\frac{\gamma_{L}}{\lambda_{L}}=\rho \cdot {SP}_{L}∙(1+kF))/kF$, $\;\frac{\gamma_{S}}{\lambda_{S}}=\rho \cdot {SP}_{S}∙(1+kF))/kF$, where *SP*_*L*_ and *SP*_*S*_ denote the slopes of the GFP-RFP curves from the two control experiments. After this reduction, we can express full-length and truncated protein concentrations in the following forms:

$$\begin{array}{l} P_{L}=\left(\gamma_{L}/\lambda_{L}\right)\cdot k_{1}\cdot m_{f}\cdot k_{3}\cdot Se\cdot T_{Total}/\left[\left(1+k_{3}\cdot Se\right)\cdot \left(1+k_{1}\cdot m_{f}\right)\right]\\ \;=\left[\frac{\rho \cdot SP_{L}\cdot \left(1+kF\right)}{kF}\right]\cdot \frac{k_{1}\cdot m_{f}\cdot k_{3}\cdot Se\cdot T_{total}}{\left[\left(1+k_{3}\cdot Se\right)\cdot \left(1+k_{1}\cdot m_{f}\right)\right]} \end{array}$$ (14)

$$\begin{array}{l} \;P_{S}=\left(\gamma_{S}/\lambda_{S}\right)\cdot k_{2}\cdot m_{f}\cdot RF\\ =\left[\frac{\rho \cdot SP_{S}\cdot \left(1+kF\right)}{kF}\right]\cdot kF\cdot m_{f} \end{array}$$ (15)

Combining Eqs 10 and 11 with Eqs 1–8, we specified the dependency of free mRNA concentration with total mRNA levels:

$$m_{f}=\frac{-\left[\left(1+kF\right)+k_{1}\left(Se\cdot T_{Total}-m_{total}\right)\right]+\sqrt{{\left[\left(1+kF\right)+k_{1}\left(Se\cdot T_{Total}-m_{total}\right)\right]}^{2}+4\cdot k_{1}\left(1+kF\right)m_{total}}}{2k_{1}\left(1+kF\right)}$$ (16)

With *m*_*f*_, we can express *P*_*L*_ and *P*_*S*_ in analytic forms. Hence, the function of *P*_*total*_ with respect to *m*_*total*_ can be established.

### A parameter estimation algorithm

We developed a grid-search algorithm to find the parameter values that best fit the experimental data. Among the six undetermined parameters, ρ_p_ is an arbitrary parameter that only affects the scale of selenoprotein expression but not the behavior of the translation process in simulation. Thus, we first excluded ρ_p_ in the fitting algorithm and manually adjusted ρ_p_ after fitting. We generated grids with different combinations of parameters and calculated the fitness of the predicted (RFP, GFP) intensities generated by these parameters with the experimental results. The grids were first generated by logarithmically dividing each parameter into 12 intervals within their boundaries (the range of each parameter value is shown in Table 4). These parameter sets were applied to the mathematical model to convert RFP values into *P*_*L*_ and *P*_*S*_ in the loss function Q^2^:

$${TQ}^{2}=\sum_{i=1}^{n}{Q_{i}}^{2}(k_{1},\;k_{3},\;k_{f},\;T_{total},\;\rho ,\rho_{p}\;)=\sum_{i=1}^{n}{\left({P_{total}}_{i}-{P_{L}}_{i}-{P_{S}}_{i}\right)}^{2}$$

$$Q^{2}={\left\{P_{total}–\frac{\left[\frac{\rho \cdot SP_{L}\left(1+kF\right)}{kF}\right]k_{1}\cdot m_{f}\cdot k_{3}\cdot Se\cdot T_{total}}{\left[\left(1+k_{3}\cdot Se\right)\left(1+k_{1}\cdot m_{f}\right)\right]}–\left[\frac{\rho \cdot SP_{S}\left(1+kF\right)}{kF}\right]\cdot kF\cdot m_{f}\right\}}^{2}$$ (17)

The total loss function *TQ*^2^ is summed over all data points indexed by *i*. *P*_*total*_ is calculated by transforming GFP intensities using *ρ*_p_.

The loss function has a complicated nonlinear form and thus contains many local optima. Analytic algorithms such as gradient descent will likely find suboptimal solutions whose loss is far from the global minimum. We devised a variation of the divide-and-conquer heuristic approach to alleviate this problem. We started by partitioning the log-scale range of each parameter value by coarse-grained intervals. A small number of multi-dimensional grids were generated from the partitioned parameter space. We then recursively performed the following computations: (1) evaluation of loss function values of parameter configurations on the grids, (2) selection of the top 30 parameter configurations, and (3) subdivision of the selected grids into smaller intervals. Recursion stopped when the grid sizes reached the required resolution of parameter values. The criteria for selecting the parameter configuration from the top-ranking solutions are reported in S1 File. The Matlab codes of the parameter estimation algorithm are reported in S2 File. The GPS data of SEPHS2, GPX1, SEPX1, SELK and SEPW1 are reported in S3–S7 Files respectively. The top ranking solutions of the four SECIS element constructs and SEPW1 are reported in S8 File.

### Parameter estimation of simulated data

We randomly generated 100 parameter sets within each parameter boundary by the following function:

$$10^{{\text{log}}_{10}UB_{i}+\left(\log_{10}UB_{i}-\log_{10}LB_{i}\right)X}$$ (18)

Where *UB* and *LB* are the upper and lower bounds, respectively, of each parameter and *X* is a random number uniformly distributed on the open interval (0, 1). For each parameter set, about 1000 corresponding RFP and GFP values were generated by the mathematical model. The parameter estimation algorithm was applied to the simulated data, and the estimated parameter values were compared with the parameter values from which the simulated data were generated. We also introduced additive noise to the simulated data with the following formula:

$$GFP_{new}=GFP_{original}+GFP_{original}\cdot NorR$$ (19)

Where *GFP*_*original*_ denotes the GFP values calculated from the model. *NorR* is randomly drawn from a normal distribution with a mean equal to 0. The standard deviation of *NorR* varied from 0.3 to 5.0 (Table 2).

The performance of our algorithm was evaluated by the log10 ratios between predicted and underlying parameter values:

$$Error=\left|\log_{10}\frac{P_{predict}}{P_{answer}}\right|$$ (20)

Where *P*_*predict*_ denotes the parameters predicted by the algorithm and *P*_*answer*_ are the true parameters. A parameter value prediction was labeled successful if the error of at least one of the predicted parameter set was smaller than 1 among the top 15 answers reported by the algorithm. The recovering rate indicates the ratio of successful predictions among 100 test sets.

#### Parameter estimation of the experimental data

We applied the parameter estimation algorithm to about 15,000 RFP-GFP pairs measured at five selenium concentrations. For ρ_p_, we manually chose the value that yielded mRNA and protein levels within normal SEPHS2 expression ranges. We referred to MOPED [31] and BioGPS [30] to get the mRNA and protein expression levels of SEPHS2 relative to ACTN1 and ACTN2, and then converted the relative SEPHS2 expression level into absolute concentration using the dataset of absolute concentrations of ACTN1 and ACTN2 [29]. We estimated that the mRNA expression level of SEPHS2 falls within the order of 10^2^ molecules per cell and the protein expression level within 10^3^ molecules per cell.

## Supporting information

S1 Fig The GPS assay system. GPS is a dual fluorescent reporter system capable of simultaneous measurement of protein synthesis, abundance and stability in single cells [25]. In the GPS system, the reporter cassette enables translation of red fluorescent protein (RFP) and green fluorescent protein (GFP) from a single transcript via cap-dependent translation, as well as translation from the internal ribosome entry site (IRES). While RFP serves as a non-degradable internal control that reports protein synthesis, GFP is fused to the N-terminus of the protein of interest (e.g., SEPHS2) and reports protein abundance. The GFP/RFP ratio represents protein stability, measuring the relative steady-state abundance between RFP and GFP-fusion proteins. Single-cell fluorescent signals were recorded using fluorescence-activated cell sorting (FACS).
(TIF)

S2 Fig The relationship between experimental and simulated SEPHS2 expression under various selenium concentrations. Each dot denotes the GFP (proportional to total protein abundance P_T_) and RFP (proportional to total mRNA quantity) values of a single cell. Each solid circle denotes the simulated GFP value under each RFP value according to the inferred model. Yellow and orange dots denote the GPS data of mutants expressing only P_L_ and P_S_, respectively. Their (P_L_ and P_S_) mean GFP values under each RFP value are represented by solid circles of the corresponding colors.
(TIF)

S3 Fig The effect of release factor knockdown on UGA definition. Distributions of GFP/RFP ratios of P_T_ with or without shRNA-mediated knockdown of RF1.
(TIF)

S4 Fig Protein half-life and UGA definition analysis of SEPW1. (a) Protein stability measurement of P_L_ or P_S_ by the GPS assay. P_L_ and P_S_ were expressed from SEPW1 mutant transcripts that exclusively express one form of SEPW1. (b) The relationship between protein synthesis and abundance for P_L_ and P_S_ in SEPW1 analogous to Fig 2B. (c-d) GPS analysis of P_L_ and P_S_ in SEPW1 under various selenium concentrations (c) or synthesis levels (d). Relative mRNA levels represent quantifications of the RFP signals in the GPS assay.
(TIF)

S5 Fig Comparison of experimental and predicted Sec incorporation efficiencies in SEPW1. **(a)** The relationship between protein synthesis and abundance for P_T_ analyzed under five selenium concentrations from experimental data. The style follows S2 Fig. (b) The relationship between P_T_ abundance and mRNA levels under five selenium concentrations from model prediction.
(TIF)

S6 Fig Comparison of the selenoprotein hierarchy under various selenium concentrations. The relationship between protein synthesis and abundance for P_T_ analyzed under four selenium concentrations for four SECIS elements. The panels on the left column (a-d) indicate the results from experimental data. The panels on the right column (e-h) indicate the predictions from the inferred models. The selenium concentrations applied are indicated on the left.
(TIF)

S1 Table Quantitative evaluation of experimental and predicted protein abundances based on observed protein synthesis levels and selenium concentrations. (DOCX)

S2 Table Quantitative evaluation of experiment and predicted P_L_/P_S_ ratio corresponding to the relative mRNA levels from the Western blotting assay. (DOCX)

S3 Table Estimated parameter values of SEPW1. (DOCX)

S4 Table Estimated parameter values of constructs of four SECIS elements. (DOCX)

S1 File Detailed description of the data processing protocol, parameter estimation algorithm, and an augmented model for incorporating mRNA degradation. (PDF)

S2 File The Matlab codes of the parameter estimation algorithm. (ZIP)

S3 File The GPS data of SEPHS2. (ZIP)

S4 File The GPS data of the GPX1 SECIS element construct. (ZIP)

S5 File The GPS data of the SEPX1 SECIS element construct. (ZIP)

S6 File The GPS data of the SELK SECIS element construct. (ZIP)

S7 File The GPS data of SEPW1. (ZIP)

S8 File The top ranking solutions of four SECIS element constructs and SEPW1. (XLSX)

## References

[1] Lee BJ, Worland PJ, Davis JN, Stadtman TC, Hatfield DL. Identification of a selenocysteyl-tRNA(Ser) in mammalian cells that recognizes the nonsense codon, UGA. The Journal of biological chemistry. 1989;264(17):9724–7. 2498338

[2] Srinivasan G, James CM, Krzycki JA. Pyrrolysine encoded by UAG in Archaea: charging of a UAG-decoding specialized tRNA. Science. 2002;296(5572):1459–62. 10.1126/science.1069588 12029131

[3] Lin H-C, Ho S-C, Chen Y-Y, Khoo K-H, Hsu P-H, Yen H-CS. CRL2 aids elimination of truncated selenoproteins produced by failed UGA/Sec decoding. Science. 2015;349(6243):91–5. 10.1126/science.aab0515 26138980 PMC4766860

[4] Driscoll DM, Copeland PR. Mechanism and regulation of selenoprotein synthesis. Annual review of nutrition. 2003;23:17–40. 10.1146/annurev.nutr.23.011702.073318 12524431

[5] Hatfield DL, Gladyshev VN. How Selenium Has Altered Our Understanding of the Genetic Code. Molecular and Cellular Biology. 2002;22(11):3565–76. 10.1128/MCB.22.11.3565-3576.2002 11997494 PMC133838

[6] Papp LV, Lu J, Holmgren A, Khanna KK. From selenium to selenoproteins: synthesis, identity, and their role in human health. Antioxidants & redox signaling. 2007;9(7):775–806. Epub 2007/05/19. 10.1089/ars.2007.1528 17508906

[7] Allmang C, Krol A. Selenoprotein synthesis: UGA does not end the story. Biochimie. 2006;88(11):1561–71. 10.1016/j.biochi.2006.04.015 16737768

[8] Low SC, Grundner-Culemann E, Harney JW, Berry MJ. SECIS-SBP2 interactions dictate selenocysteine incorporation efficiency and selenoprotein hierarchy. The EMBO journal. 2000;19(24):6882–90. 10.1093/emboj/19.24.6882 11118223 PMC305907

[9] Copeland PR, Driscoll DM. Purification, redox sensitivity, and RNA binding properties of SECIS-binding protein 2, a protein involved in selenoprotein biosynthesis. The Journal of biological chemistry. 1999;274(36):25447–54. 10464275 10.1074/jbc.274.36.25447

[10] Copeland PR, Fletcher JE, Carlson BA, Hatfield DL, Driscoll DM. A novel RNA binding protein, SBP2, is required for the translation of mammalian selenoprotein mRNAs. The EMBO journal. 2000;19(2):306–14. 10.1093/emboj/19.2.306 10637234 PMC305564

[11] Tujebajeva RM, Copeland PR, Xu XM, Carlson BA, Harney JW, Driscoll DM, et al Decoding apparatus for eukaryotic selenocysteine insertion. EMBO Rep. 2000;1(2):158–63. 10.1093/embo-reports/kvd033 11265756 PMC1084265

[12] Berry MJ, Tujebajeva RM, Copeland PR, Xu XM, Carlson BA, Martin GW 3rd, et al Selenocysteine incorporation directed from the 3'UTR: characterization of eukaryotic EFsec and mechanistic implications. Biofactors. 2001;14(1–4):17–24. 11568436 10.1002/biof.5520140104

[13] Nakamura Y, Ito K, Isaksson LA. Emerging understanding of translation termination. Cell. 1996;87(2):147–50. 8861897 10.1016/s0092-8674(00)81331-8

[14] Kisselev L, Ehrenberg M, Frolova L. Termination of translation: interplay of mRNA, rRNAs and release factors? The EMBO journal. 2003;22(2):175–82. 10.1093/emboj/cdg017 12514123 PMC140092

[15] Suppmann S, Persson BC, Bock A. Dynamics and efficiency in vivo of UGA-directed selenocysteine insertion at the ribosome. The EMBO journal. 1999;18(8):2284–93. 10.1093/emboj/18.8.2284 10205181 PMC1171311

[16] Kollmus H, Flohe L, McCarthy JE. Analysis of eukaryotic mRNA structures directing cotranslational incorporation of selenocysteine. Nucleic acids research. 1996;24(7):1195–201. 8614619 10.1093/nar/24.7.1195 PMC145795

[17] Mehta A, Rebsch CM, Kinzy SA, Fletcher JE, Copeland PR. Efficiency of mammalian selenocysteine incorporation. The Journal of biological chemistry. 2004;279(36):37852–9. 10.1074/jbc.M404639200 15229221 PMC2820281

[18] Berry MJ, Harney JW, Ohama T, Hatfield DL. Selenocysteine insertion or termination: factors affecting UGA codon fate and complementary anticodon:codon mutations. Nucleic acids research. 1994;22(18):3753–9. 7937088 10.1093/nar/22.18.3753 PMC308358

[19] Howard MT, Carlson BA, Anderson CB, Hatfield DL. Translational redefinition of UGA codons is regulated by selenium availability. The Journal of biological chemistry. 2013;288(27):19401–13. 10.1074/jbc.M113.481051 23696641 PMC3707644

[20] Jameson RR, Diamond AM. A regulatory role for Sec tRNA[Ser]Sec in selenoprotein synthesis. RNA. 2004;10(7):1142–52. 10.1261/rna.7370104 15208449 PMC1370604

[21] Latreche L, Jean-Jean O, Driscoll DM, Chavatte L. Novel structural determinants in human SECIS elements modulate the translational recoding of UGA as selenocysteine. Nucleic acids research. 2009;37(17):5868–80. 10.1093/nar/gkp635 19651878 PMC2761289

[22] Low SC, Harney JW, Berry MJ. Cloning and functional characterization of human selenophosphate synthetase, an essential component of selenoprotein synthesis. The Journal of biological chemistry. 1995;270(37):21659–64. 7665581 10.1074/jbc.270.37.21659

[23] Schomburg L, Schweizer U. Hierarchical regulation of selenoprotein expression and sex-specific effects of selenium. Biochimica et biophysica acta. 2009;1790(11):1453–62. 10.1016/j.bbagen.2009.03.015 19328222

[24] Kryukov GV, Castellano S, Novoselov SV, Lobanov AV, Zehtab O, Guigo R, et al Characterization of mammalian selenoproteomes. Science. 2003;300(5624):1439–43. 10.1126/science.1083516 12775843

[25] Yen HC, Xu Q, Chou DM, Zhao Z, Elledge SJ. Global protein stability profiling in mammalian cells. Science. 2008;322(5903):918–23. 10.1126/science.1160489 18988847

[26] Mansell JB, Guevremont D, Poole ES, Tate WP. A dynamic competition between release factor 2 and the tRNA(Sec) decoding UGA at the recoding site of Escherichia coli formate dehydrogenase H. The EMBO journal. 2001;20(24):7284–93. 10.1093/emboj/20.24.7284 11743004 PMC125778

[27] Baker KE, Parker R. Nonsense-mediated mRNA decay: terminating erroneous gene expression. Curr Opin Cell Biol. 2004;16(3):293–9. 10.1016/j.ceb.2004.03.003 15145354

[28] Chang YF, Imam JS, Wilkinson MF. The nonsense-mediated decay RNA surveillance pathway. Annual review of biochemistry. 2007;76:51–74. 10.1146/annurev.biochem.76.050106.093909 17352659

[29] Schwanhausser B, Busse D, Li N, Dittmar G, Schuchhardt J, Wolf J, et al Global quantification of mammalian gene expression control. Nature. 2011;473(7347):337–42. 10.1038/nature10098 21593866

[30] Wu C, Orozco C, Boyer J, Leglise M, Goodale J, Batalov S, et al BioGPS: an extensible and customizable portal for querying and organizing gene annotation resources. Genome biology. 2009;10(11):R130 10.1186/gb-2009-10-11-r130 19919682 PMC3091323

[31] Kolker E, Higdon R, Haynes W, Welch D, Broomall W, Lancet D, et al MOPED: Model Organism Protein Expression Database. Nucleic acids research. 2012;40(Database issue):D1093–9. 10.1093/nar/gkr1177 22139914 PMC3245040

[32] Fletcher JE, Copeland PR, Driscoll DM, Krol A. The selenocysteine incorporation machinery: interactions between the SECIS RNA and the SECIS-binding protein SBP2. RNA. 2001;7(10):1442–53. 11680849 PMC1370188

[33] Ding F, Grabowski PJ. Identification of a protein component of a mammalian tRNA(Sec) complex implicated in the decoding of UGA as selenocysteine. RNA. 1999;5(12):1561–9. 10606267 10.1017/s1355838299991598 PMC1369878

[34] Xu XM, Mix H, Carlson BA, Grabowski PJ, Gladyshev VN, Berry MJ, et al Evidence for direct roles of two additional factors, SECp43 and soluble liver antigen, in the selenoprotein synthesis machinery. The Journal of biological chemistry. 2005;280(50):41568–75. 10.1074/jbc.M506696200 16230358

[35] Moran U, Phillips R, Milo R. SnapShot: key numbers in biology. Cell. 2010;141(7):1262–e1. 10.1016/j.cell.2010.06.019 20603006

[36] Xu XM, Carlson BA, Mix H, Zhang Y, Saira K, Glass RS, et al Biosynthesis of selenocysteine on its tRNA in eukaryotes. PLoS biology. 2007;5(1):e4 10.1371/journal.pbio.0050004 17194211 PMC1717018

[37] Wolfe RR, Song J, Sun J, Zhang XJ. Total aminoacyl-transfer RNA pool is greater in liver than muscle in rabbits. The Journal of nutrition. 2007;137(11):2333–8. 17951466 10.1093/jn/137.11.2333

[38] Iben JR, Maraia RJ. tRNA gene copy number variation in humans. Gene. 2014;536(2):376–84. 10.1016/j.gene.2013.11.049 24342656 PMC3941035

[39] Dittmar KA, Goodenbour JM, Pan T. Tissue-specific differences in human transfer RNA expression. PLoS genetics. 2006;2(12):e221 Epub 2006/12/30. 10.1371/journal.pgen.0020221 17194224 PMC1713254

[40] King MP, Attardi G. Post-transcriptional regulation of the steady-state levels of mitochondrial tRNAs in HeLa cells. The Journal of biological chemistry. 1993;268(14):10228–37. 7683672

[41] Lee HW, Ryu JY, Yoo J, Choi B, Kim K, Yoon TY. Real-time single-molecule coimmunoprecipitation of weak protein-protein interactions. Nature protocols. 2013;8(10):2045–60. 10.1038/nprot.2013.116 24071910

[42] Boisvert FM, Ahmad Y, Gierlinski M, Charriere F, Lamont D, Scott M, et al A quantitative spatial proteomics analysis of proteome turnover in human cells. Molecular & cellular proteomics: MCP. 2012;11(3):M111 011429. Epub 2011/09/23. 10.1074/mcp.M111.011429 21937730 PMC3316722

[43] Donovan J, Copeland PR. Selenocysteine insertion sequence binding protein 2L is implicated as a novel post-transcriptional regulator of selenoprotein expression. PLoS One. 2012;7(4):e35581 10.1371/journal.pone.0035581 22530054 PMC3328465

