# Supplementary figures and images for: A quantitative model for the rate-limiting process of UGA alternative assignments to stop and selenocysteine codons
Source: PLoS Comput Biol. 2017 Feb 8;13(2):e1005367. doi: 10.1371/journal.pcbi.1005367 (PMC5323020; doi:10.1371/journal.pcbi.1005367)

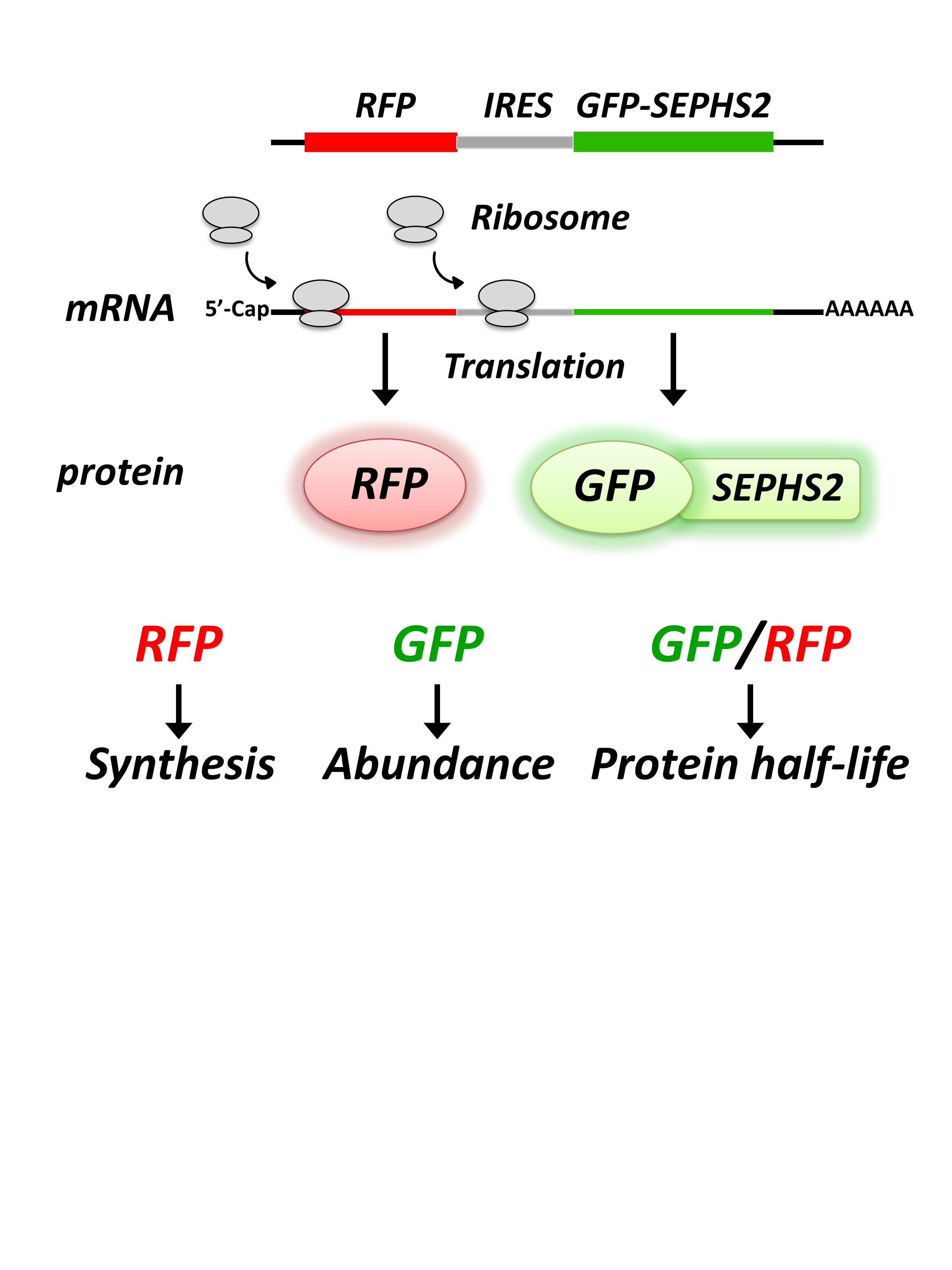

Supplement: S1 Fig — GPS is a dual fluorescent reporter system capable of simultaneous measurement of protein synthesis, abundance and stability in single cells [25]. In the GPS system, the reporter cassette enables translation of red fluorescent protein (RFP) and green fluorescent protein (GFP) from a single transcript via cap-dependent translation, as well as translation from the internal ribosome entry site (IRES). While RFP serves as a non-degradable internal control that reports protein synthesis, GFP is fused to the N-terminus of the protein of interest (e.g., SEPHS2) and reports protein abundance. The GFP/RFP ratio represents protein stability, measuring the relative steady-state abundance between RFP and GFP-fusion proteins. Single-cell fluorescent signals were recorded using fluorescence-activated cell sorting (FACS). (TIF) [file pcbi.1005367.s001.TIF]

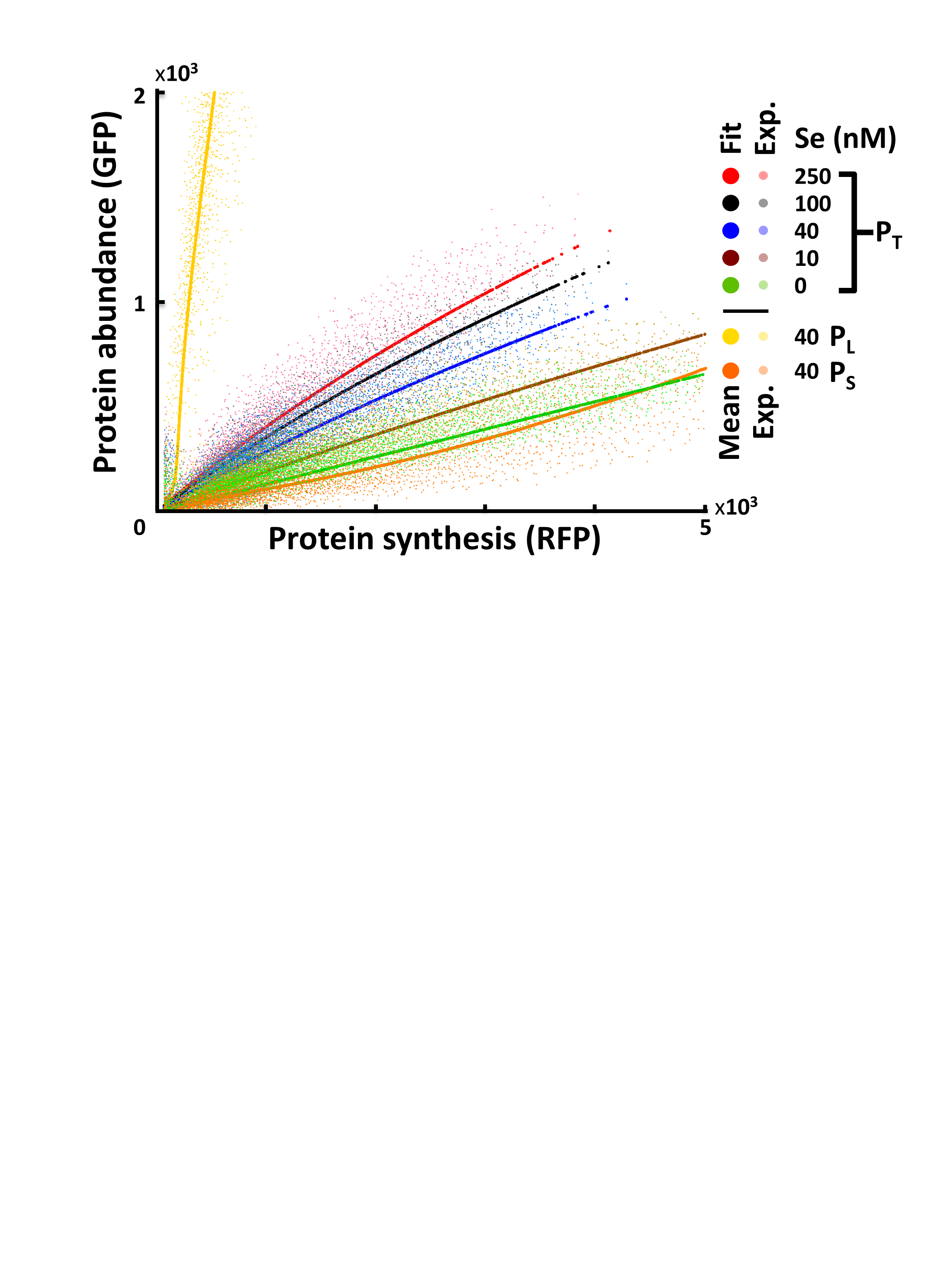

Supplement: S2 Fig — Each dot denotes the GFP (proportional to total protein abundance PT) and RFP (proportional to total mRNA quantity) values of a single cell. Each solid circle denotes the simulated GFP value under each RFP value according to the inferred model. Yellow and orange dots denote the GPS data of mutants expressing only PL and PS, respectively. Their (PL and PS) mean GFP values under each RFP value are represented by solid circles of the corresponding colors. (TIF) [file pcbi.1005367.s002.TIF]

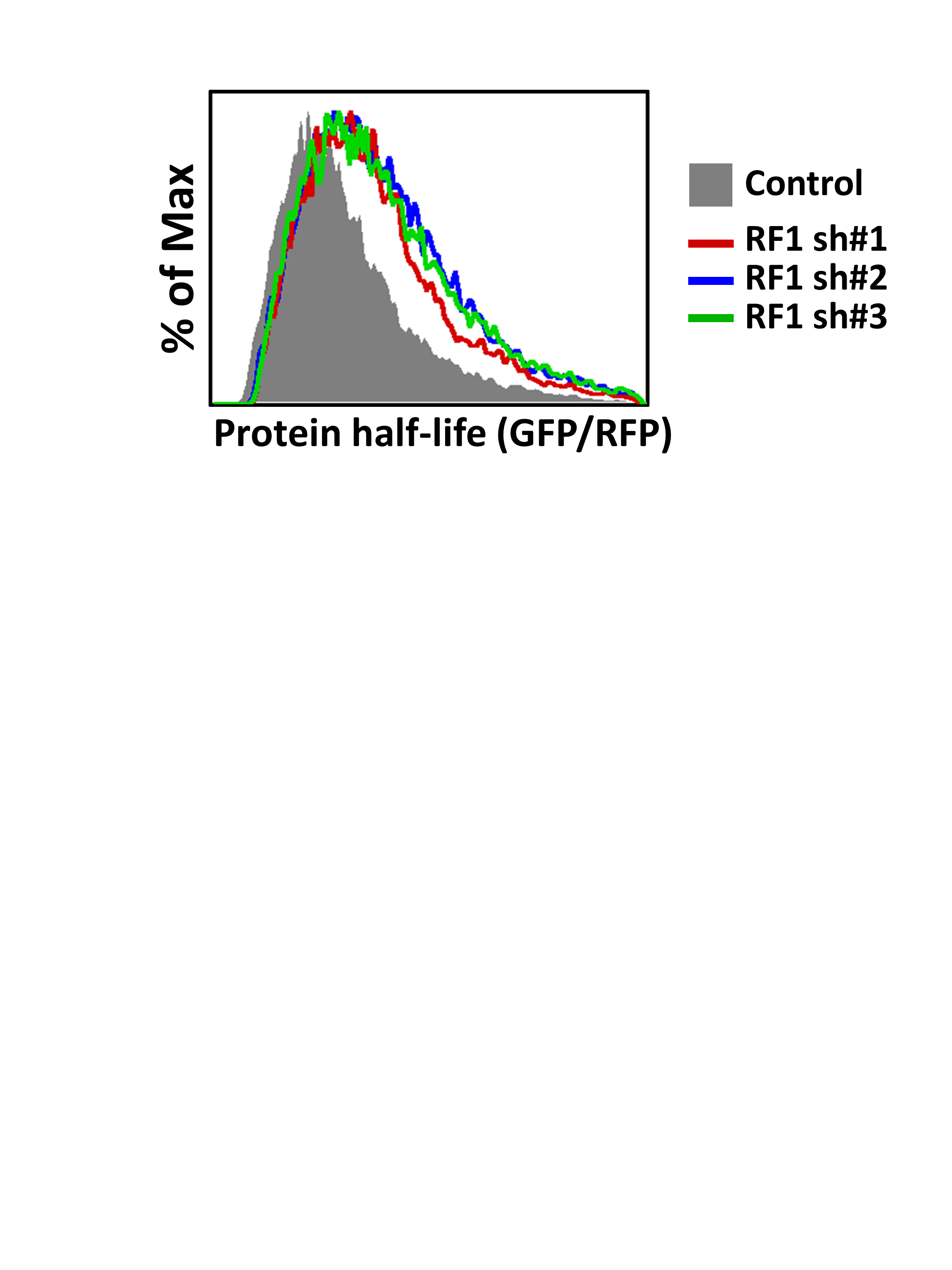

Supplement: S3 Fig — Distributions of GFP/RFP ratios of PT with or without shRNA-mediated knockdown of RF1. (TIF) [file pcbi.1005367.s003.TIF]

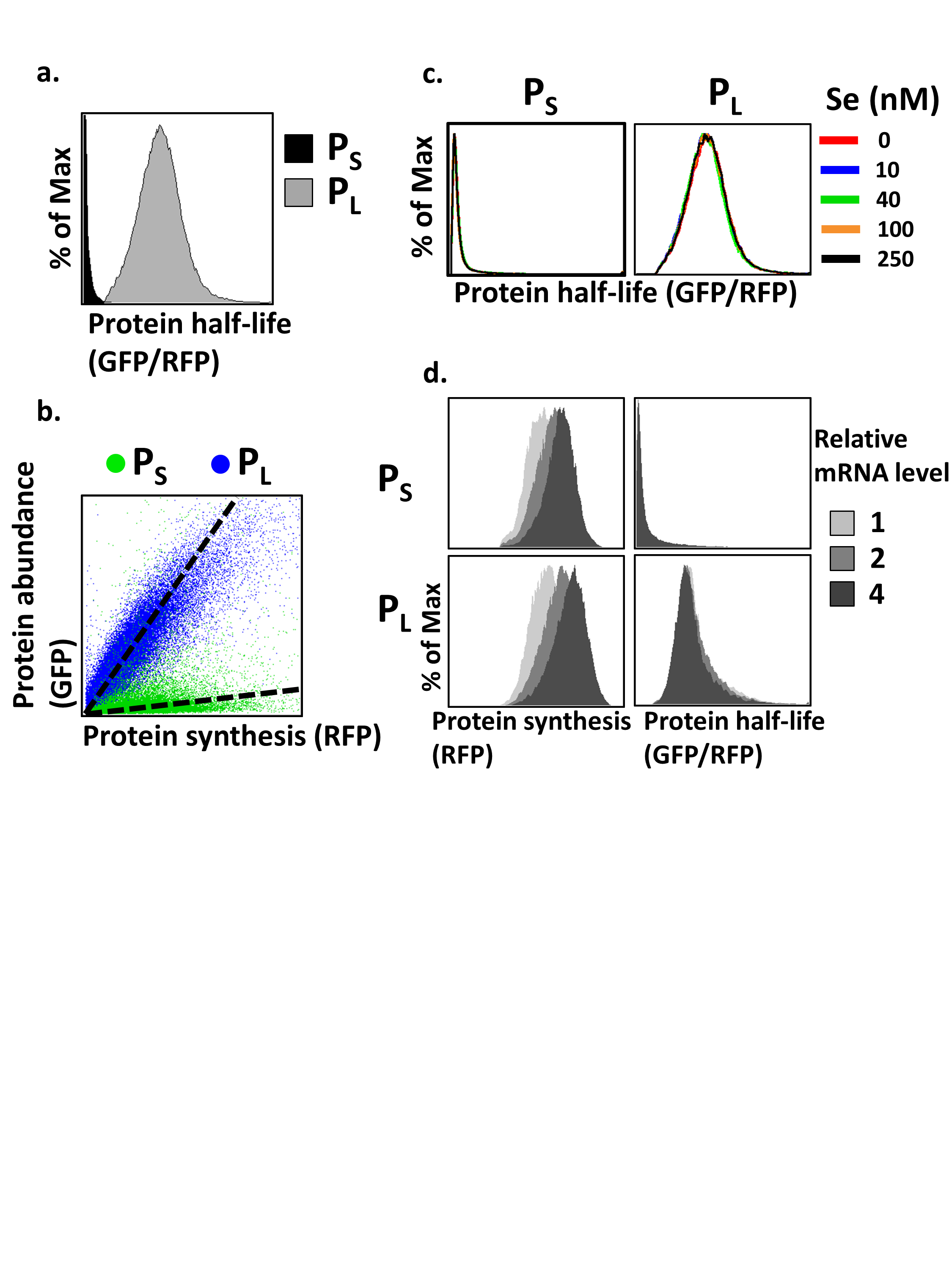

Supplement: S4 Fig — (a) Protein stability measurement of PL or PS by the GPS assay. PL and PS were expressed from SEPW1 mutant transcripts that exclusively express one form of SEPW1. (b) The relationship between protein synthesis and abundance for PL and PS in SEPW1 analogous to Fig 2B. (c-d) GPS analysis of PL and PS in SEPW1 under various selenium concentrations (c) or synthesis levels (d). Relative mRNA levels represent quantifications of the RFP signals in the GPS assay. (TIF) [file pcbi.1005367.s004.TIF]

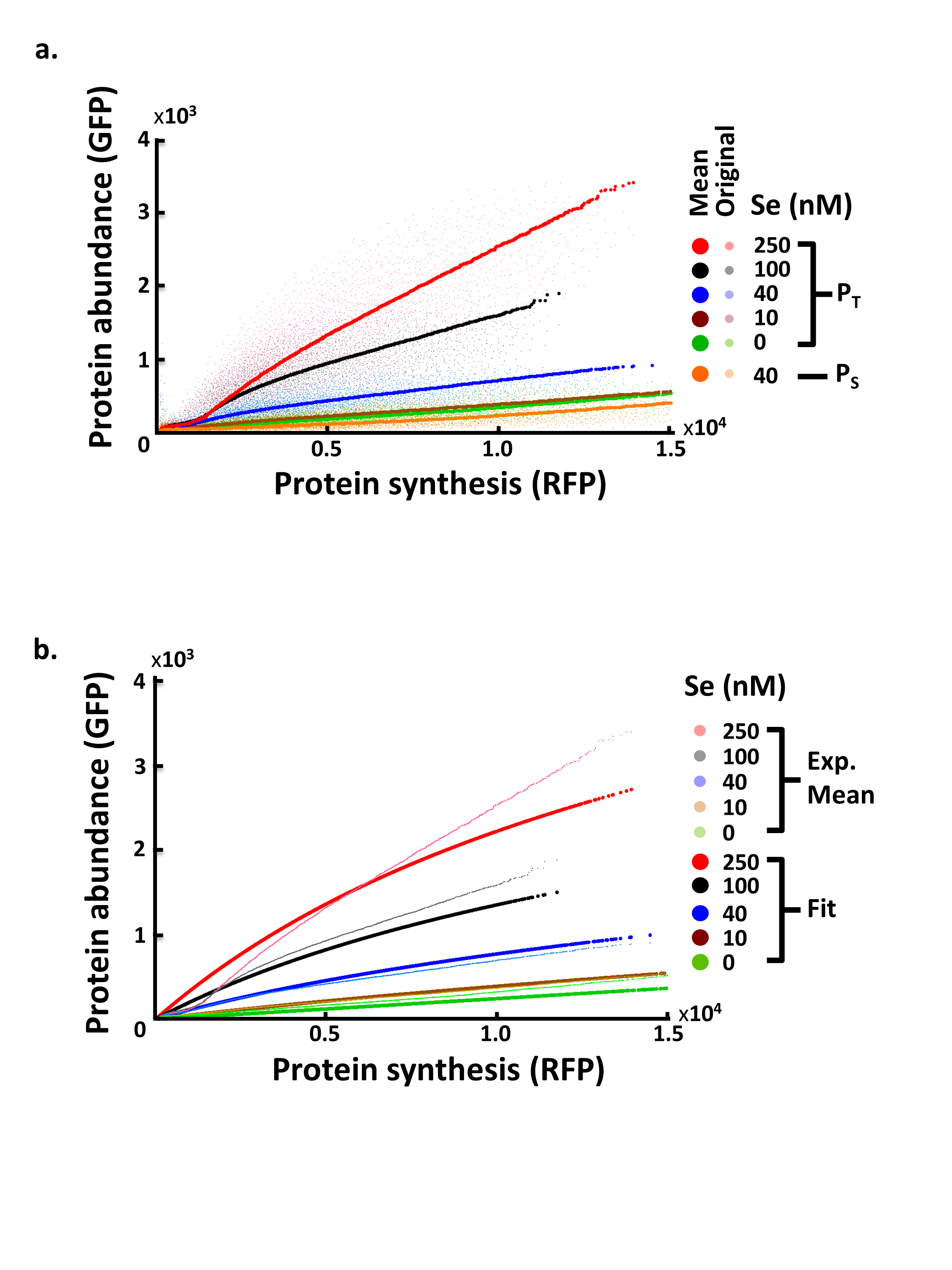

Supplement: S5 Fig — (a) The relationship between protein synthesis and abundance for PT analyzed under five selenium concentrations from experimental data. The style follows S2 Fig. (b) The relationship between PT abundance and mRNA levels under five selenium concentrations from model prediction. (TIF) [file pcbi.1005367.s005.TIF]

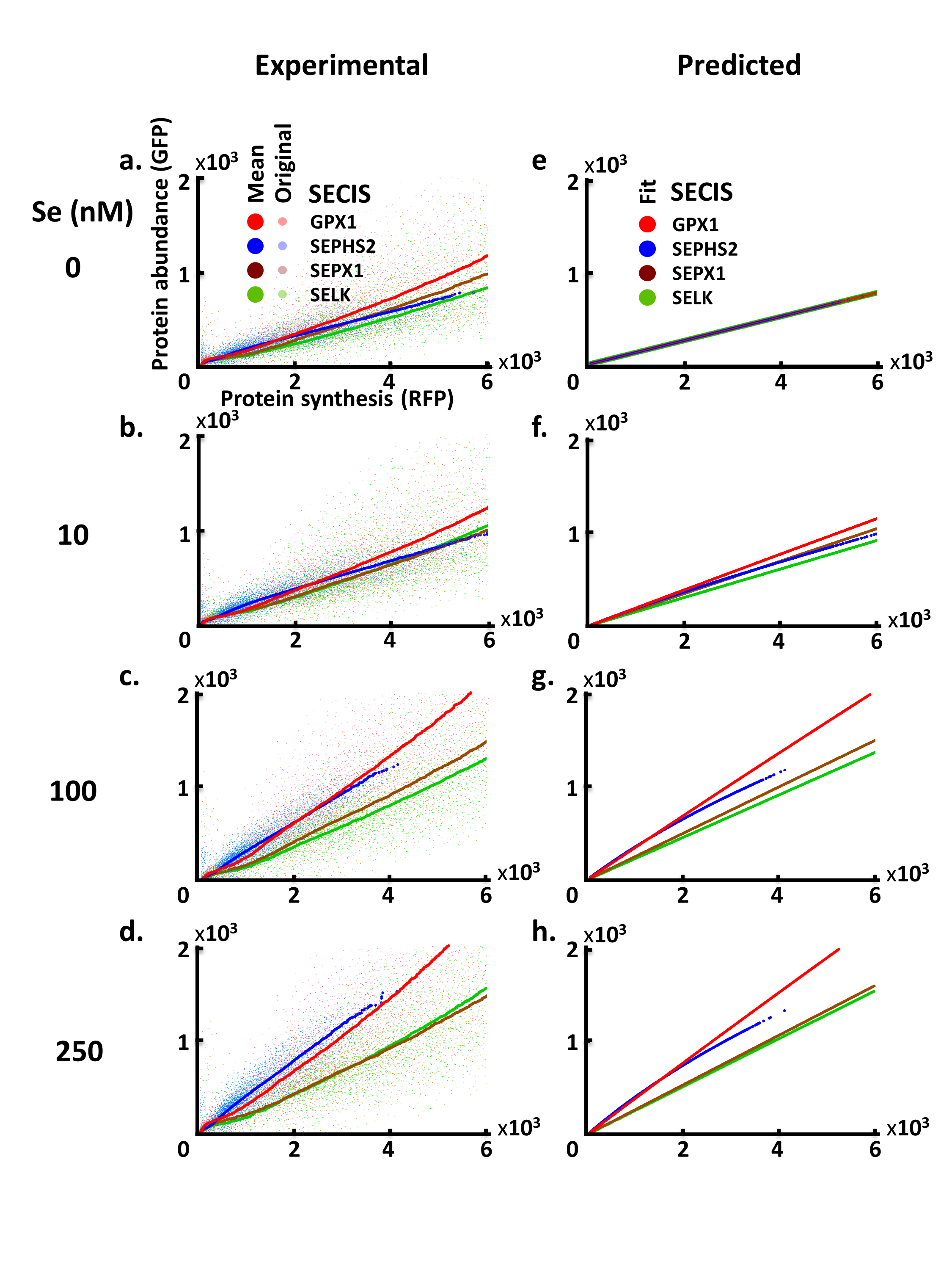

Supplement: S6 Fig — The relationship between protein synthesis and abundance for PT analyzed under four selenium concentrations for four SECIS elements. The panels on the left column (a-d) indicate the results from experimental data. The panels on the right column (e-h) indicate the predictions from the inferred models. The selenium concentrations applied are indicated on the left. (TIF) [file pcbi.1005367.s006.TIF]
