# Supplementary material for: A quantitative model for the rate-limiting process of UGA alternative assignments to stop and selenocysteine codons
Source: PLoS Comput Biol. 2017 Feb 8;13(2):e1005367. doi: 10.1371/journal.pcbi.1005367 (PMC5323020; doi:10.1371/journal.pcbi.1005367)
Supplement: S1 Table — (DOCX) [file pcbi.1005367.s007.docx]

**S1 Table. Quantitative evaluation of experimental and predicted protein abundances based on observed protein synthesis levels and selenium concentrations.**

| **Evaluation** | **Constraint Model** | **Selenium concentrations (nM)** | | | | |
| --- | --- | --- | --- | --- | --- | --- |
|  |  | **0** | **10** | **40** | **100** | **250** |
| *r^2^* | mRNA-tRNA | 0.9970 | 0.9986 | 0.9994 | 0.9972 | 0.9975 |
|  | mRNA | 0.9970 | 0.9988 | 0.9992 | 0.9958 | 0.9982 |
|  | tRNA | 0.9970 | 0.9956 | 0.9959 | 0.9995 | 0.9992 |
| RMSE | mRNA-tRNA | 4.62E+01 | 1.78E+01 | 2.25E+01 | 2.05E+01 | 3.78E+01 |
|  | mRNA | 4.62E+01 | 2.53E+01 | 1.40E+02 | 5.40E+02 | 1.27E+03 |
|  | tRNA | 2.75E+06 | 3.01E+06 | 1.96E+06 | 1.89E+06 | 1.77E+06 |

Two quantitative scores – *r^2^* and root mean square error – are reported for three models and five selenium concentrations. The models with the mRNA constraint yield superior fit to the data.
