# Supplementary material for: A quantitative model for the rate-limiting process of UGA alternative assignments to stop and selenocysteine codons
Source: PLoS Comput Biol. 2017 Feb 8;13(2):e1005367. doi: 10.1371/journal.pcbi.1005367 (PMC5323020; doi:10.1371/journal.pcbi.1005367)
Supplement: S2 Table — (DOCX) [file pcbi.1005367.s008.docx]

**S2 Table. Quantitative evaluation of experiment and predicted P_L_/P_S_ ratio corresponding to the relative mRNA levels from the Western blotting assay.**

| **Evaluation** | **Constraint Model** | **Simulated mRNA levels** | | | |
| --- | --- | --- | --- | --- | --- |
|  |  | **1000** | **2100** | **3300** | **4400** |
| *r^2^* | mRNA-tRNA | 0.386 | 0.440 | 0.883 | 0.880 |
|  | mRNA | 0.000 | 0.012 | 0.328 | 0.573 |
|  | tRNA | 0.390 | 0.442 | 0.883 | 0.880 |
| RMSE | mRNA-tRNA | 2.522 | 0.311 | 0.133 | 0.077 |
|  | mRNA | 11.414 | 7.623 | 5.060 | 3.836 |
|  | tRNA | 3.489 | 0.796 | 0.543 | 0.401 |

The models with the tRNA constraint yield superior fit to the data.
