# Supplementary material for: A quantitative model for the rate-limiting process of UGA alternative assignments to stop and selenocysteine codons
Source: PLoS Comput Biol. 2017 Feb 8;13(2):e1005367. doi: 10.1371/journal.pcbi.1005367 (PMC5323020; doi:10.1371/journal.pcbi.1005367)
Supplement: S3 Table — (DOCX) [file pcbi.1005367.s009.docx]

**S3 Table. Estimated parameter values of SEPW1.**

| ***k_1_*** | ***kF*** | ***k_3_*** | ***T_total_*** | ***ρ*** | ***ρ_p_*** | ***Q^2^*** |  |
| --- | --- | --- | --- | --- | --- | --- | --- |
| 13.13 | 5814.48 | 0.003 | 100.00 | 64.87 | 0.45 | 4.09E+09 | |
