# Supplementary material for: A quantitative model for the rate-limiting process of UGA alternative assignments to stop and selenocysteine codons
Source: PLoS Comput Biol. 2017 Feb 8;13(2):e1005367. doi: 10.1371/journal.pcbi.1005367 (PMC5323020; doi:10.1371/journal.pcbi.1005367)
Supplement: S4 Table — (DOCX) [file pcbi.1005367.s010.docx]

**S4 Table. Estimated parameter values of constructs of four SECIS elements.**

| **SECIS element** | ***k_1_*** | ***kF*** | ***k_3_*** | ***T_total_*** | ***ρ*** | ***ρ_p_*** | ***Q^2^*** |
| --- | --- | --- | --- | --- | --- | --- | --- |
| SEPHS2 | 17.03 | 9803.03 | 0.021 | 63.44 | 10.40 | 0.95 | 1.48E+08 |
| GPX1 | 12.20 | 8521.31 | 0.026 | 60.47 | 177.83 | 1.00 | 2.71E+09 |
| SEPX1 | 5.73 | 9803.03 | 0.040 | 71.88 | 10000.00 | 0.80 | 4.17E+09 |
| SELK | 5.73 | 8522.76 | 0.016 | 65.74 | 10000.00 | 1.00 | 1.72E+09 |
