# Supplementary material for: A quantitative model for the rate-limiting process of UGA alternative assignments to stop and selenocysteine codons
Source: PLoS Comput Biol. 2017 Feb 8;13(2):e1005367. doi: 10.1371/journal.pcbi.1005367 (PMC5323020; doi:10.1371/journal.pcbi.1005367)
Supplement: S1 File — (PDF) [file pcbi.1005367.s011.pdf]

Content of Supplementary File S1

1. **Criteria of estimated parameter selection** 2
2. **Derivation of the mRNA accuracy rates** 2

1. Criteria of estimated parameter selection

The grid search algorithm reports multiple estimated parameters to experimental data under given ρ_p. The parameters are sorted by loss function Q^2, and top 5 combinations are retrieved for each given ρ_p. The best estimated parameter values to fit experimental data have to satisfy the following criteria.

1. For each ρ_p value, select the five parameter combinations that give the lowest Q^2. Rule out the ρ_p values with substantially higher average Q^2 than others.
2. Among the remaining ρ_p values, select the ones with the lowest degeneracy of parameters k_1, kF, k_3 and T_{total}.
3. kF and T_{total} values inferred from the four SECIS elements – SEPHS2, GPX1, SEPX1, SELK – are similar.

Q^2 represents the fitness of estimated parameters. In the meanwhile, the lower degeneracy of top combinations also reflects the overall fitness of given ρ_p. Degeneracy is assessed by the coefficient of variation (CV%) for {k_1, kF, k_3, T_{total}}. Since quantities of release factor and $tRNA^{sec}$ are invariant in the four SECIS constructs, kF and T_{total} should be similar among them. Therefore, the consensus of kF and T_{total} is considered as an additional criteria.

We searched parameter values on six-dimensional grids, where boundary values of the grids were determined according physiologically reasonable ranges. The upper boundary of six parameters, {k_1, kF, k_3, T_{total}, ρ, ρ_p } is {1e2, 1e4, 1e2, 1e3, 1e4, 1e4}. The lower boundary setting is {1e-3, 1e-3, 1e-3, 1e-1, 1e-3, 1e-3}. The intervals between the low and high boundaries in each parameter are equally dissected into 12 subintervals. We evaluated the Q^2 on the grids and selected the parameter configurations according to criteria 1-3. Top solutions are in Table 4, Table S3 and supporting file 8, except for GPX1. The GPX1 solution obtained from the aforementioned search range and criteria has

excessively lower *kF* than other genes. To enforce all solutions satisfy criteria 3, we narrowed the search ranges of {*kF*, T_{total}} to be {1e4, 80} and {7e3, 60}, respectivly. After searching by refined grid, we obtain the final solutions and summaried as Table 4 and supporting file 8.

2. Derivation of the mRNA accuracy rates

Denote m, S_1 and S_2 the concentrations of free mRNAs, charged tRNAs, and release factors, and mS_1, mS_2 as the concentrations of mRNAs bound by tRNA or RF. The rates of mRNA binding with tRNAs and RFs are:

$$\begin{aligned}\frac{dmS_1}{dt}&=k_1S_1m.\\ \frac{dmS_2}{dt}&=k_2S_2m.\end{aligned} \tag{1}$$

Within a fixed short time interval, the probabilities of mRNA binding with tRNA and RF are linearly related to k_1S_1 and k_2S_2 respectively.

We establish the following assumptions:

1. At the beginning of each time interval, a fixed amount of f_0 mRNAs are generated.
2. Each mRNA binds to tRNA, RF or none with probabilities $p_1, p_2, 1-p_1-p_2$ respectively.
3. Each mRNA possesses a state variable denoting the number of synthesis cycles it already undergoes.
4. If an mRNA undergoes no synthesis cycle before and binds to RF, then it is degraded with certainty.

5. If an mRNA undergoes N synthesis cycles, then it is degraded with certainty.

6. Otherwise, an mRNA degrades with probability $e \ll 1$.

Denote c the state variable of the number of synthesis cycles an mRNA already undergoes. We derive the probabilities $P(c=0), P(c=1), \cdots, P(c=N)$ as follows.

At time step t, denote $n_0(t)$, $n_1(t)$, …, $n_N(t)$ the number of mRNA molecules that already undergo 0, 1, $\cdots N$ synthesis cycles. At time step t+1, there are the following changes:

1. The mRNAs with c=0 include the old mRNAs that do not bind with tRNAs and RFs, and newly supplied mRNAs.

2. The mRNAs with c=1 include the old mRNAs with c=1 at time t and do not bind with tRNAs and RFs in this cycle, and the old mRNAs with c=0 at time t and bind with tRNAs.

3. The mRNAs with $c=k$, $k>1$ include the old mRNAs with $c=k$ at time t and do not bind with tRNAs and RFs in this cycle, and the old mRNAs with $c=k-1$ at time t and bind with tRNAs or RFs.

4. The mRNAs with $c=N$ become degraded.

We can write those relations in a system of difference equations:

$$
\begin{aligned}
n_0(t+1) &= n_0(t)(1-p_1-p_2)+f_0 \\
n_1(t+1) &= n_0(t)p_1+n_1(t)(1-p_1-p_2) \\
n_2(t+1) &= n_1(t)(p_1+p_2)+n_2(t)(1-p_1-p_2) \\
\cdots & \quad \cdots\cdots \\
n_N(t+1) &= n_{N-1}(t)(p_1+p_2)+n_N(t)(1-p_1-p_2)
\end{aligned} \tag{2}
$$

The steady state solution of these equations is

$$
\begin{aligned}
n_0 &= \frac{f_0}{p_1+p_2} \\
n_1 &= \frac{p_1}{p_1+p_2} n_0 \\
n_2 &= n_1 \\
&\cdots \cdots\cdots \\
n_N &= n_{N-1}
\end{aligned} \tag{3}
$$

The probability of $P(c=k)= \frac{n_k}{n_0+\cdots+n_N}$. Thus

$$
\begin{aligned}
P(c=0) &= \frac{1}{(N-1)\eta+1} \\
P(c=1) &= \frac{\eta}{(N-1)\eta+1} \\
\cdots \quad & \cdots\cdots \\
P(c=N) &= \frac{\eta}{(N-1)\eta+1}
\end{aligned} \tag{4}
$$

$\eta= \frac{p_1}{p_1+p_2}$. Since p_1 and p_2 are linearly related to k_1S_1 and k_2S_2, $\eta= \frac{k_1S_1}{k_1S_1+k_2S_2}$.

For mRNAs binding with tRNAs, $P(decay)=e_0$.

For mRNAs bindings with RFs,

$$
\begin{aligned}
P(decay) &= \sum_{k=0}^{N} P(c=k)P(decay|c=k) \\
&= P(c=0)\cdot 1+P(c>0)\cdot e_0 \\
&= \frac{1}{(N-1)\eta+1}+\frac{(N-1)\eta}{(N-1)\eta+1}\cdot e_0
\end{aligned} \tag{5}
$$

Thus, the accuracy rates of long and short forms are

$$\alpha_L = 1 - e_0$$
$$\alpha_S = 1 - \frac{1}{(N-1)\eta + 1} - \frac{(N-1)\eta}{(N-1)\eta + 1} \cdot e_0 \qquad (6)$$

respectively.
