# Supplementary material for: A quantitative model for the rate-limiting process of UGA alternative assignments to stop and selenocysteine codons
Source: PLoS Comput Biol. 2017 Feb 8;13(2):e1005367. doi: 10.1371/journal.pcbi.1005367 (PMC5323020; doi:10.1371/journal.pcbi.1005367)
Supplement: S2 File — (ZIP) [file pcbi.1005367.s012.zip › README.rtf]

A Quantitative Model for the Rate-Limiting Process of UGA Alternative Assignments to Stop and Selenocysteine Codons

This section is the MATLAB codes in this study. These codes design and validate under MATLAB 2014. We separate the codes into three stages based on their functions.
1. Data pre-processing (1_DataProcess)
2. Parameter estimation of experimental data (2_ParaEstimation)
3. Generation of simulation data and corresponded curves (3_modelValidation)

We summarized all the processes into three example scripts and named with prefixed title “ToXXX” in each folder. The part of DataProcess is to process FACS csv file (1e7 signals) as reduced set (about 3e3 representative signals). The example script is “ToProcesFACS.m”. “SEPHS2_0.csv” is the example file of SEPHS2 FACS under [Se] = 0 nM. 

1.	“highDensDataFACS” generates high density data with grid size = 2000, and density >= 30.
2.	“genRandomFractionFACSData” generates random fraction set from FACS signals (Reduced to 4e-3).
3.	“uniqueMean” generates mean and statistics of given FACS signal.

The representative set is generated by following functions and named as “FACSHD3K” for next stage.

ParaEstimation is designed to search model parameters from given FACS data. The example script is “ToGradDesDivCon4_SV2_MultiRhop_12B.m”. We prepared the process data of 5 proteins into individual .mat file and name as “HD3KSe5_Protein.mat”. 

1.	“gradDesDivCon4_SV2_12B” searches the parameters by our grid search algorithm and evaluates goodness of fit, Q2.
2.	“catBestSolution” collects top solutions, { k1, kF, k3, Ttotal, rho, rhoP, Q2}, into a table ordered by Q2.

The executed result of “ToGradDesDivCon4_SV2_MultiRhop_12B.m” will generate the tops of estimated parameters of each rhoP into “topParaProtein”. We can use estimated parameters to generate simulated signals at next stage.

modelValidation is to generate simulated FACS signal based on given parameters and selenium concentrations. The example script is “ToValidateModel.m”. In the example code, we need three parts of data to generate simulated data and graph. They are mean FACS signals (FACS), pre-defined slopes (sp1 and sp2) and estimated parameters (xVec).

1.	ugaModelToFACS is for generating simulated data by given mean RFP signals, estimated parameters and specific selenium concentrations.

The example graphs of ugaModelToFACS is shown in Fig. 6a (SEPHS2) and Fig. S5a (SEPW1).
